# Clinical features and treatments of VEXAS syndrome in critical care: a scoping review

**DOI:** 10.1186/s13054-025-05390-y

**Published:** 2025-04-17

**Authors:** Kasumi Satoh, Yasushi Tsujimoto, Daisuke Kasugai, Kazuki Okura, Sarah Kyuragi Luthe, Takao Ono, Yuki Miyamoto, Tasuku Matsuyama, Manabu Okuyama, Taketo Watase, Hajime Nakae, Tadahiro Goto

**Affiliations:** 1 Department of Emergency and Critical Care Medicine, Akita University Graduate School of Medicine, 1 - 1- 1 Hondo, Akita, 010 - 8543 Japan; 2 Department of Health Promotion and Human Behavior, Kyoto University Graduate School of Medicine/School of Public Health, Kyoto, Japan; 3 Oku Medical Clinic, Osaka, Japan; 4 Scientific Research WorkS Peer Support Group (SRWS-PSG), Osaka, Japan; 5 Department of Emergency and Critical Care Medicine, Nagoya University Graduate School of Medicine, Nagoya, Japan; 6 Division of Rehabilitation, Akita University Hospital, Akita, Japan; 7 Department of Anesthesiology and Critical Care Medicine, Asahikawa Medical University, Asahikawa, Japan; 8 Carus Medical Group, Tokyo, Japan; 9 Department of Emergency and General Internal Medicine, Fujita Health University, Toyoake, Japan; 10 Department of Emergency Medicine, Kyoto Prefectural University of Medicine, Kyoto, Japan; 11 Department of Health Data Science, Yokohama City University, Kanagawa, Japan; 12 GOTO Research Co. Ltd., Tokyo, Japan; 13 TXP Medical Co. Ltd., Tokyo, Japan

**Keywords:** Intensive care units, X-linked genetic diseases, Critical care, Sepsis, Multiple organ failure

## Abstract

**Background:**

Vacuoles, E1 enzyme, X-linked, autoinflammatory, somatic (VEXAS) syndrome is a recently discovered severe disorder that predominantly affects adult males, characterized by systemic inflammation and hematologic abnormalities. Despite its profound impact on patient outcomes, awareness of VEXAS syndrome among critical care providers remains severely limited, often leading to delayed recognition, diagnosis, and initiation of appropriate treatment. This study aims to address this knowledge gap by conducting a scoping review on VEXAS syndrome in the critical care setting.

**Methods:**

This scoping review followed the PRISMA-ScR guidelines and Joanna Briggs Institute methodology, analyzing data from Cochrane CENTRAL, MEDLINE via PubMed, EMBASE, and Web of Science on May 19, 2024. We included studies that reported clinical features and treatments of patients with VEXAS syndrome requiring critical care.

**Results:**

Of the 1262 reports identified, 78 reports met the inclusion criteria, including 45 case reports/series, 17 observational studies, 15 reviews, and one systematic review. Analysis of 55 cases revealed a median age of 69 with a strong male predominance (54/55). ICU admission rates ranged from 28 to 33%, with mortality rates between 18 and 40%. Critical manifestations included shock, hemophagocytic lymphohistiocytosis, acute respiratory distress syndrome, thrombosis, and airway edema. Sepsis was the leading cause of death, followed by other causes including VEXAS syndrome related organ failure, cardiovascular events, and intestinal perforation. Treatment approaches combined conventional critical care measures with immunosuppressive and immunomodulatory therapies, although infectious complications were frequently reported.

**Conclusion:**

This review revealed the lack of systematically analyzed studies focusing on VEXAS syndrome in the critical care setting, suggesting a significant gap in understanding the clinical characteristics and optimal treatments for VEXAS syndrome. Further research focused on VEXAS syndrome in the critical care setting is essential to improve early recognition, develop standardized treatment protocols, and ultimately improve patient outcomes in this complex patient population.

**Supplementary Information:**

The online version contains supplementary material available at 10.1186/s13054-025-05390-y.

## Introduction

Vacuoles, E1 enzyme, X-linked, autoinflammatory, somatic (VEXAS) syndrome is a newly identified, late-onset autoinflammatory and hematological disorder first described by Beck et al. in 2020 [1]. VEXAS syndrome is caused by somatic mutations in the ubiquitin-like modifier activating enzyme 1 (UBA1) gene located on the X chromosome, predominantly affecting adult males, and characterized by a variety of clinical manifestations. These clinical manifestations have been categorized into two main features: systemic inflammation and those affecting the hematologic system [1]. Clinically, VEXAS syndrome exhibits a wide range of systemic inflammatory manifestations, including fever, skin rashes, arthritis, chondritis of the ear and nose, pulmonary infiltrates, ocular inflammation, and an increased risk of venous thromboembolism [2]. The hallmark hematologic manifestation of VEXAS syndrome is the presence of cytoplasmic vacuoles in myeloid and erythroid precursor cells within the bone marrow, often associated with myelodysplastic syndromes [2].

Given the severe inflammation, organ dysfunction, and thrombophilia, management of VEXAS syndrome is highly challenging, as some patients develop life-threatening organ failure requiring critical care and intensive care unit (ICU) admission [3, 4]. Indeed, this syndrome is reported with high morbidity and mortality. Initial reports indicated that up to 40% of patients died [1], and subsequent studies have reported mortality rates as high as 50% [5].

Despite the critical nature of VEXAS syndrome and the potential need for intensive care management, there is no comprehensive summary of evidence focusing on this disease in the critical care settings. VEXAS syndrome is a recently identified condition with diverse and overlapping clinical manifestations, often leading to misdiagnosis and inappropriate treatment, which can negatively affect patient outcomes. Furthermore, limited access to genetic testing remains a significant challenge in its diagnosis and management. Consequently, it is crucial to consolidate existing knowledge on the presentation and management on this syndrome in the critical care setting. Therefore, this study aims to address the knowledge gap on VEXAS syndrome by conducting a scoping review on its presentation and management in the critical care setting, summarizing current clinical characteristics and treatment approaches, and highlighting areas that require further research.

## Methods

### Scoping review

A scoping review was conducted in accordance with a pre-published protocol from the Protocol.io database [6] with reference to current review methodologies based on the Preferred Reporting Items for Systematic Reviews and Meta-Analysis extension for Scoping Reviews (PRISMA-ScR) guidelines and Joanna Briggs Institute (JBI) [7–9].

### Research question and eligibility criteria

This study aims to address the following review questions. (1) What are the clinical characteristics of VEXAS syndrome in critical care settings? and (2) What are the current treatment approaches for VEXAS syndrome in critical care settings? To explore the clinical characteristics, we also reviewed cases where VEXAS syndrome mimicked other diseases, potentially leading to misdiagnosis or delayed diagnosis in the critical care setting. Furthermore, we investigated treatment approaches including definitive treatments, supportive therapy, and life-sustaining interventions.

Patient eligibility criteria are as follows: patients diagnosed with VEXAS syndrome who required or were expected to require critical care, including those with high severity or needing ICU admission. There were no restrictions on patient location (e.g., general ward, ICU), region, race, or gender. The publication date of the literature was not limited, and only English-language publications were included. We included a wide range of study designs: experimental and quasi-experimental (e.g., randomized and non-randomized controlled trials, before-and-after studies, interrupted time-series analyses), analytical observational (e.g., cohort, case–control, cross-sectional), descriptive observational (e.g., case series, case reports), qualitative research, systematic and narrative reviews, opinion papers, and conference proceedings. Given the recent identification of VEXAS syndrome, we employed a broad search approach to include studies with various publication dates and publication statuses, ensuring the comprehensive inclusion of all relevant literature on this topic.

### Search strategy, selection of studies, and data extraction

We searched the Cochrane Central Register of Controlled Trials (CENTRAL), MEDLINE via PubMed, EMBASE, and Web of Science on May 19, 2024. The full search strategy for these databases was developed using terms and keywords appearing in the titles and abstracts of relevant articles and the index terms used to describe the articles (see Supplementary Table 1). After the search, all identified citations were uploaded to Rayyan (Rayyan, Massachusetts, USA), and duplicates were removed. Two reviewers (KS and KO) independently screened the titles and abstracts of the search results to determine whether each citation met the inclusion criteria. Subsequently, eligibility was evaluated through an independent full-text review conducted by the same reviewers. Any disagreements between reviewers at each stage of the selection process were resolved through discussion or by consulting an additional reviewer (TG). When further information was required, we contacted the authors of the extracted studies. Data were extracted for all included studies using a previously defined and agreed data extraction protocol which comprised information about the author, year of publication, study design, study purpose, population, sample size, study methodology, clinical information, and key findings relevant to the scoping review questions. The content corresponding to the key findings relevant to the scoping review questions were determined by the same evaluating reviewers including a board-certified critical care physician (KS) and medical staff (KO) with the Japanese Society of Intensive Care Medicine. For example, we identified acute conditions that resulted in death, conditions treated in the ICU, and descriptions of clearly fatal risks. For the extraction process, two reviewers independently identified relevant data by marking the text within the reference documents. One reviewer then compiled these marked data into a final spreadsheet, which both reviewers subsequently verified for accuracy and refined through consensus on the final dataset.

### Data analysis and presentation

We conducted a structured analysis of the extracted data focusing on critical care aspects of VEXAS syndrome. Data synthesis was organized into two main categories: (1) clinical characteristics and (2) treatment approaches. For relevant cases, we systematically extracted patient demographics, clinical manifestations, treatment approaches, and outcomes. Data from observational studies were analyzed separately, focusing on ICU admission rates and mortality. To visualize the relationships between clinical manifestations and outcomes, we created an UpSet plot showing the co-occurrence of major clinical features across reported cases. Key clinical characteristics and patient outcomes were summarized in a table. Additionally, we developed clinical course charts to illustrate the typical disease trajectories of patients who became critically ill prior to the diagnosis of VEXAS syndrome.

## Results

### Characteristics of included studies

**Fig. 1:**
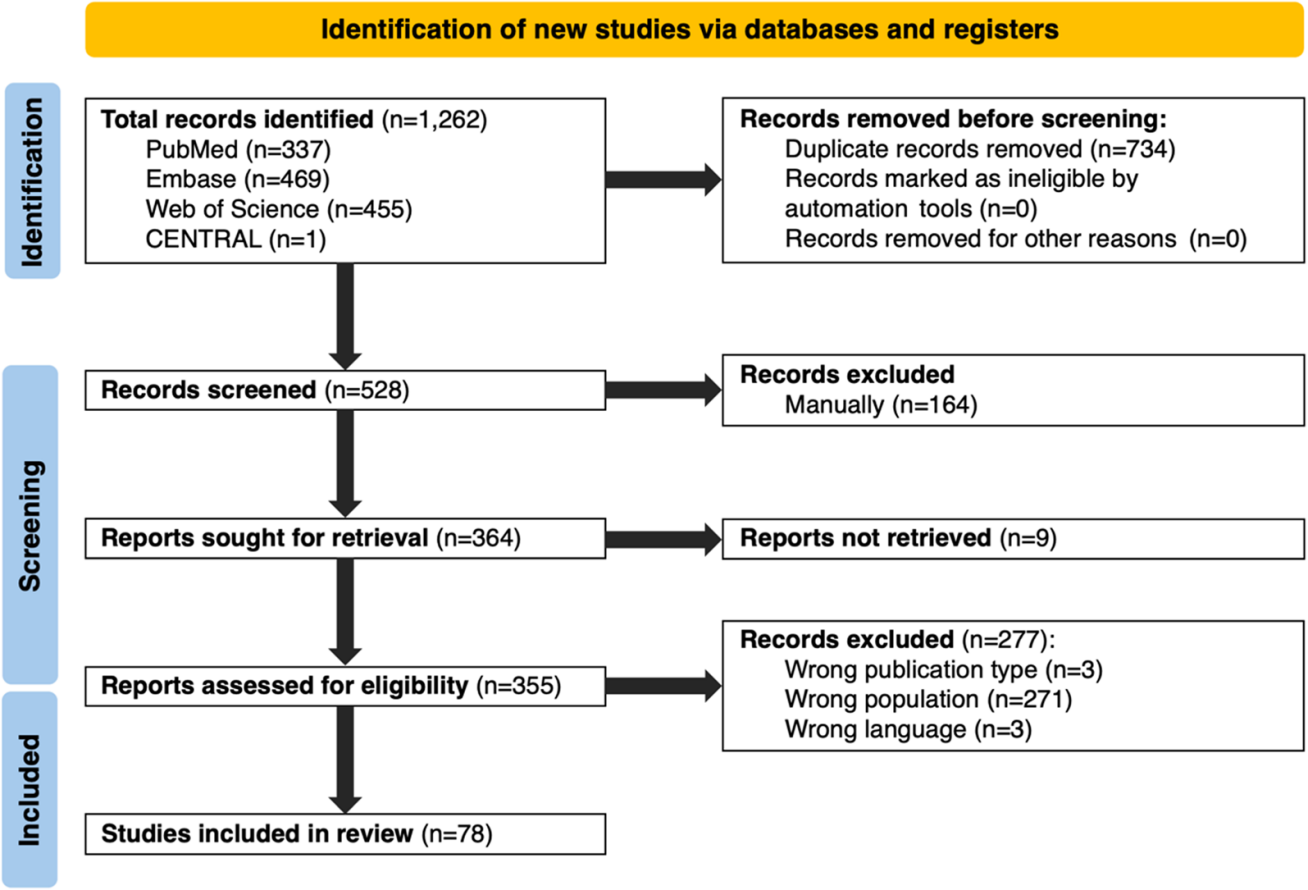
Flow diagram of study selection. The flow diagram illustrates the literature search and study selection process. Initially, 1262 records were identified through database searches. After removing 734 duplicates, 528 records remained for screening. Of the 355 reports assessed for eligibility, 277 were excluded due to publication type (n = 3), population mismatch (n = 271), or language restrictions (n = 3). Finally, 78 studies were included in the review. This flow diagram adheres to the Preferred Reporting Items for Systematic Reviews and Meta-Analyses (PRISMA) 2020 guidelines for reporting systematic reviews

A total of 1262 reports were identified. After removing duplicates and conducting screening of titles, abstracts, and full-text reviews, 78 reports met the inclusion criteria (Fig. 1). These included 45 case reports or case series, 17 analytical observational studies, 15 narrative reviews, and 1 systematic review. Of note, 10 of the 78 reports were conference papers. Publication years ranged from 2020 to 2024. Of these studies including patients with VEXAS syndrome, one case report explicitly focused on intensive or critical care settings [3], in addition to two studies including some ICU patient data [10, 11]. Two reviewers initially agreed on inclusion/exclusion of 304 out of 355 reports (85.6% agreement). A third reviewer was not required as the two reviewers reached full consensus on all study inclusions following a detailed discussion.

### Clinical manifestations leading to critical states

**Fig. 2:**
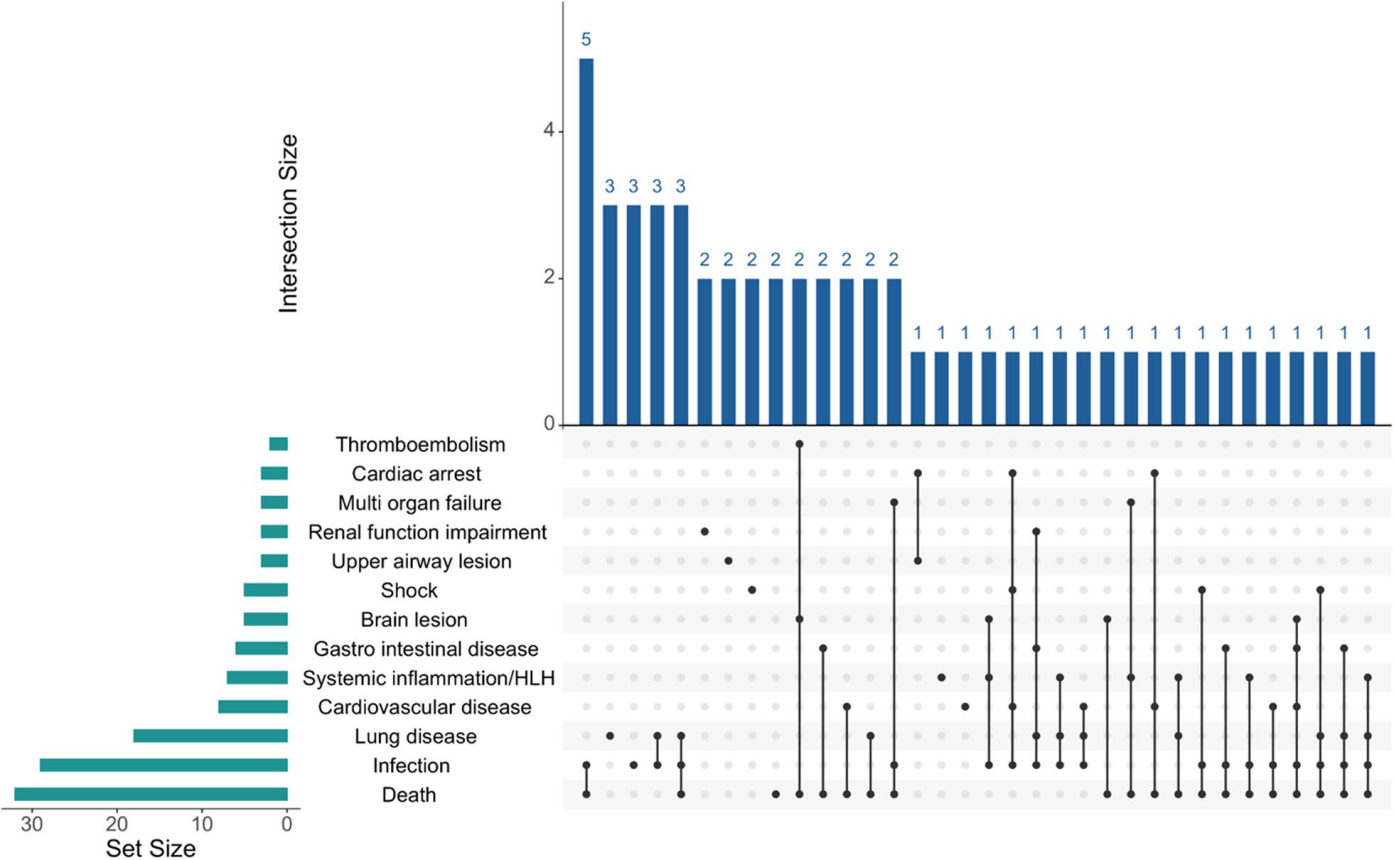
UpSet plot illustrating the frequency and overlap of symptoms among 55 cases of VEXAS syndrome in the critical care setting. The horizontal green bars on the left (Set Size) indicate the number of patients who experienced each individual complication. The vertical blue bars at the top (Intersection Size) represent the number of patients with the specific combination of complications, as shown by the connected black dots in the matrix below. Each row in the matrix corresponds to a particular complication, and black dots connected by lines denote co-occurring complications in a subset of patients. The numerical values above each vertical bar indicate the size of these subsets. This figure highlights patterns of co-occurring complications—such as infection, thromboembolism, and lung disease—in critically ill patients with VEXAS syndrome. *HLH* Hemophagocytic lymphohistiocytosis, *VEXAS* Vacuoles, E1 enzyme, X-linked, Autoinflammatory, Somatic mutations

From the included literature, a total of 55 cases of VEXAS syndrome relevant to critical care were identified and extracted (Supplemental Table 5). The reported patients were predominantly male (54/55), with a median age of 69 years (range: “late 30 s” per documented in report up to 82). Nine patients were explicitly noted to have received intensive care; however, this does not necessarily imply that the others did not receive critical care or treatment in the ICU. In observational studies, reported ICU admissions proportion ranged from 28 to 33%. Approximately two-thirds (32/55) died in individual case reports, and the reported mortality varied across analytical observational studies, ranging from 18 to 40%, with follow-up periods typically ranging between 30 months and 4.4 years. Sepsis was the leading cause of death, and other causes included organ failure, cardiovascular events, and intestinal perforation (Table 1). While shock and hyperinflammatory states in VEXAS syndrome can be severe and often require critical care, five cases with shock were reported. Of these, three had septic shock, one had anaphylactic shock, and one was reported as distributive shock. Additionally, one case involved overlapping septic and cardiogenic shock. Furthermore, four patients developed hemophagocytic lymphohistiocytosis (HLH), likely driven by dysregulated macrophage activation [12]. Excessive inflammation was also associated with acute tubulointerstitial nephritis, encephalitis [13–15], and intestinal perforation in three cases, two linked to tocilizumab [5, 16]. Figure 2 illustrates the distribution of major clinical manifestations across each reported case report (n = 55). Infections were frequently observed and commonly coexisted with death. However, the figure also showed that various other critical conditions co-occurred with death.

**Table 1:** Clinical characteristics of severe VEXAS syndrome based on data from analytical observational studies, including the types and incidences of significant complications and mortality

Author and year	Country	Aim	Population	Sample size	Key findings relevant to the scoping review questions
Borie et al. [46]	France	To provide more detailed information about lung disease in patients with VEXAS syndrome	Patients with VEXAS syndrome included retrospectively in a national database in France	N = 51	Respiratory insufficiency: 11.8% (n = 6)
Comont et al. [47]	France	To describe the efficacy and safety of azacitidine	Myelodysplastic syndromes treated with azacitidine, included in a nationwide VEXAS syndrome cohort in France, and retrospectively diagnosed as VEXAS syndrome	N = 11	Serious adverse events: Pneumocystis infection (n = 1), severe colitis and bacterial pneumonia (n = 1) Deaths: Infection after bone marrow transplant (n = 1)
Moura et al. [8]	United States	To characterize lung involvement in VEXAS syndrome	All patients with VEXAS syndrome evaluated at Mayo Clinic in the United States	N = 45	Mortality: 8 patients died (18%, median 30 months follow-up) Causes of death: Pneumonia (2 COVID- 19, 2 bacterial), heart failure (n = 1), sepsis (n = 1), unknown (n = 2)
Ferrada et al. [10]	United States	To define the prevalence of VEXAS syndrome within the relapsing polychondritis cohort; to compare clinical features between relapsing polychondritis patients with and without VEXAS syndrome; to create a clinical algorithm to identify VEXAS syndrome among relapsing polychondritis	All patients enrolled in the National Institutes of Health Relapsing Polychondritis cohort, plus additional UBA1 mutation-positive patients meeting relapsing polychondritis criteria from other National Institutes of Health cohorts and a hospital in the United Kingdom	N = 13	Intensive care unit admission: 33% (n = 4)
Valence et al. [11, 48]	France	To describe serious infectious complications and their potential risk factors	Patients aged 18 or older with confirmed VEXAS syndrome from 40 centers in France, with available data on serious infections and at least one year of follow-up since initial VEXAS symptoms	N = 124	133 serious infections occurred in 74 patients Common infection sites: lung (59%), skin (10%), urinary tract (9%), bloodstream (9%) Microbiological confirmation obtained in 76% of cases: bacterial (52%), viral (30%), fungal (15%), and mycobacterial (3%) Pulmonary infection pathogens: severe acute respiratory syndrome coronavirus 2 (28%), Legionella pneumophila (21%), Pneumocystis jirovecii (19%) Non-immunosuppressed patients: 16% of serious infections, a high proportion of atypical infections (Legionella pneumophila and Pneumocystis jirovecii); this suggests VEXAS syndrome may represent an acquired immune deficiency with susceptibility to specific pathogens Risk factors for serious infections: age > 75 years, p.Met41 Val mutation, arthralgia, Janus kinase inhibitors Mortality: 27 patients died (36%, median 4.4 years follow-up), 15 deaths (56%) due to serious infections Intensive care unit admission: 28% Surgery required: 5%
Georgin-Lavialle et al. [20]	France	To describe the clinical presentation and laboratory features of VEXAS syndrome; to determine clinical and prognostic phenotypes; to analyze phenotype-genotype correlations, overall survival, and factors associated with death	VEXAS syndrome in France with confirmed UBA1 mutations, identified through national laboratories and clinical networks	N = 116	Deaths: 18 patients (15.5%, median 3.0 years follow-up) Causes of death: infectious origin (9 cases; 7 bacterial, 2 COVID- 19), myelodysplastic syndromes progression (3 cases), cardiovascular events (2 cases), other causes (4 cases) Factors associated with death: gastrointestinal involvement, lung infiltrates, mediastinal lymph node enlargement
Comont et al. [49]	France	To determine the efficacy, safety, and prognostic factors of azacitidine treatment	Typical VEXAS syndrome from a nationwide registry of France with UBA1 mutations who received at least one full cycle of azacitidine	N = 57	Deaths: 16/57 patients (median 29 months follow-up) Causes of death while on azacitidine: 1 COVID- 19, 3 other infections, 1 VEXAS, 3 unrelated Serious adverse events during azacitidine treatment: 30 (53%) patients Most common serious adverse event: Infections (n = 25) Deaths: 16 patients died (28%, median 29 months follow-up) Causes of death while on azacitidine: COVID- 2019 (n = 1), other infections (n = 3), VEXAS syndrome (n = 1), unrelated (n = 3) Serious adverse events during azacitidine treatment: 53% Most common serious adverse event: infections (n = 25)
Kusne et al. [50]	United States	To characterize the timeline and impact of hematologic manifestations of VEXAS syndrome	Patients with confirmed VEXAS syndrome in Mayo Clinic in the United States	N = 38	Deaths: 23% (n = 9) Cause of death: infection (n = 4), cardiovascular disease (n = 1), stroke (n = 1), unknown cause (n = 3)
Gurnari et al. [51]	Italia	To document the current diagnostic capabilities and clinical-genomic features of VEXAS syndrome; to track UBA1 longitudinal clonal dynamics upon different therapeutics	VEXAS syndrome from the original cohort collected nationwide by the Italian Society of Experimental Hematology and the Italian Society of Rheumatology	N = 41	Overall survival at 1 year: 95% Deaths: 5 patients (all with p.Met41 Thr genotype) Causes of death: infectious complications (n = 3), spontaneous bowel perforation (n = 1), disease progression to acute myeloid leukemia (n = 1)
Alcedo Andrade et al. [52]	United Kingdom	To describe the incidence and characteristics of thrombosis with VEXAS syndrome	Patients with confirmed VEXAS syndrome from two cohorts: National Institutes of Health Clinical Center and the referring institutions in the United Kingdom	N = 86	Myocardial infarction, 8% (n = 7); stroke, 2% (n = 2)
Heiblig et al. [53]	International	To characterize safety efficacy profiles of Janus kinase inhibitors	VEXAS syndrome treated with different Janus kinase inhibitors	N = 30	Most frequent adverse events: infections (36.7%), thromboembolic complications (20%) Deaths: 10% (n = 3) Causes of death: legionellosis (n = 1, with tofacitinib), colon cancer progression (n = 1, with ruxolitinib), undetermined cause (n = 1, with ruxolitinib)
Beck et al. [1]	United States and United Kingdom	To identify the genetic cause of inflammatory disease	Patients with somatic UBA1 variants from National Institutes of Health genetic databases, National Institutes of Health Clinical Center cohorts, and United Kingdom hospitals	N = 25	Deaths: 40% (n = 10) Causes of death: VEXAS syndrome-related causes (respiratory failure or progressive anemia), complications related to treatment
Gutierrez-Rodrigues et al. [54]	United States	To define the clonal hematopoiesis landscape and its impact in a large cohort of patients with VEXAS syndrome using error-corrected and single-cell DNA sequencing and correlate these findings with clinical outcomes	VEXAS syndrome from the Mayo Clinic cohort in the United States and the National Institutes of Health cohort in the United Kingdom	N = 80	Overall survival at 10 years: 60% Most frequent causes of death: infections, uncontrolled inflammation
Kusne et al. [55]	United States	To describe the incidence and characteristics of thrombosis in VEXAS syndrome, correlate its presence with clinical and survival outcomes, and assess for potential risk factors	Confirmed VEXAS syndrome from the Mayo Clinic cohort in the United States	N = 119	Venous thrombosis events: 49 patients total, pulmonary embolism (n = 17, 35%) Arterial thrombosis events: 15 patients total, Stroke (n = 5, 33%); myocardial infarction (n = 7, 47%); other arterial events (n = 3, 1 critical limb ischemia and 2 acute lower limb thrombosis); multiple arterial events (n = 1: myocardial infarction and splenic infarction) Factors associated with worse survival: age, pulmonary involvement Deaths: n = 25 Reported causes of death (n = 14): infection (n = 6), progression of VEXAS syndrome (n = 4) No deaths due to thrombosis One patient with intracranial hemorrhage (on rivaroxaban with mild thrombocytopenia)
Vitale et al. [56]	International	To characterize orbital or ocular involvement in VEXAS syndrome; to identify associations with other disease features	VEXAS syndrome from the Autoinflammatory Disease Alliance international cohort	N = 59	Mortality: 10% (n = 6, median disease duration until death of 1.2 years) Causes of death: intestinal perforation (n = 1), acute respiratory failure (n = 2), infection (n = 1), unreported cause
Heiblig et al. [57]	International	To report the clinical efficacy of Janus kinase inhibitors in VEXAS syndrome	VEXAS patient treated with Janus kinase inhibitors	N = 24	Lethal legionellosis with tofacitinib (n = 1)

This table summarizes information on the authors and publication year, study aims, patient populations, sample sizes, and key findings related to significant complications, infections, mortality, and other clinically relevant outcomes with VEXAS syndrome within the critical care setting

*COVID- 19* coronavirus disease 2019; UBA1, Ubiquitin-like modifier activating enzyme 1; VEXAS, Vacuoles, E1 enzyme, X-linked, Autoinflammatory, Somatic mutations

In four of the reports, VEXAS syndrome was initially misclassified as other severe conditions in critical care settings. Patients were initially diagnosed with adult-onset Still’s disease or HLH [5, 17, 18]. Another case presenting with distributive shock was first treated as septic shock until VEXAS syndrome was later confirmed [19].

The main clinical features of VEXAS syndrome, which are not limited to the critical care setting, included lung involvement, thrombosis, and chondritis [20]. This review found that these clinical features lead to critical states in patients with VEXAS syndrome. The details on each clinical feature leading to critical conditions are discussed below.

#### Lung involvement

Severe lung complications other than lung infection were reported in 5 of 55 cases. These included two cases of acute respiratory distress syndrome (ARDS), one case of interstitial pneumonia, one case of diffuse alveolar hemorrhage, and one case of unspecified pulmonary complications.

#### Thrombosis

Significant thrombosis was reported in three cases: cerebral sinus vein thrombosis in two cases and bilateral pulmonary embolism in one case. While venous thromboembolism was commonly reported in VEXAS syndrome in previous studies [21], direct life-threatening cases of pulmonary embolism requiring shock treatment or mechanical circulatory or respiratory support were not detected in our review.

#### Chondritis

Some cases reported polychondritis affecting the airway. Three cases of upper airway edema associated with chondritis were reported, of which one included prominent supraglottic larynx edema, and two other cases of retro-cricoarytenoid edema associated with cardiac arrest and subglottic edema.

These three clinical features including lung involvement, thrombosis, and chondritis may represent key indicators of VEXAS syndrome which could aid in the diagnosis of this underrecognized condition.

### The diagnostic trajectory of severe VEXAS syndrome

We identified cases that progressed to a critical state prior to the diagnosis of VEXAS syndrome [3, 4, 13, 14, 17, 19, 22–39] and characterized the disease course from onset to the development of severe manifestations and eventual diagnosis. By exploring disease trajectories, this review equips readers with the knowledge to effectively diagnose VEXAS syndrome.

**Fig. 3:**
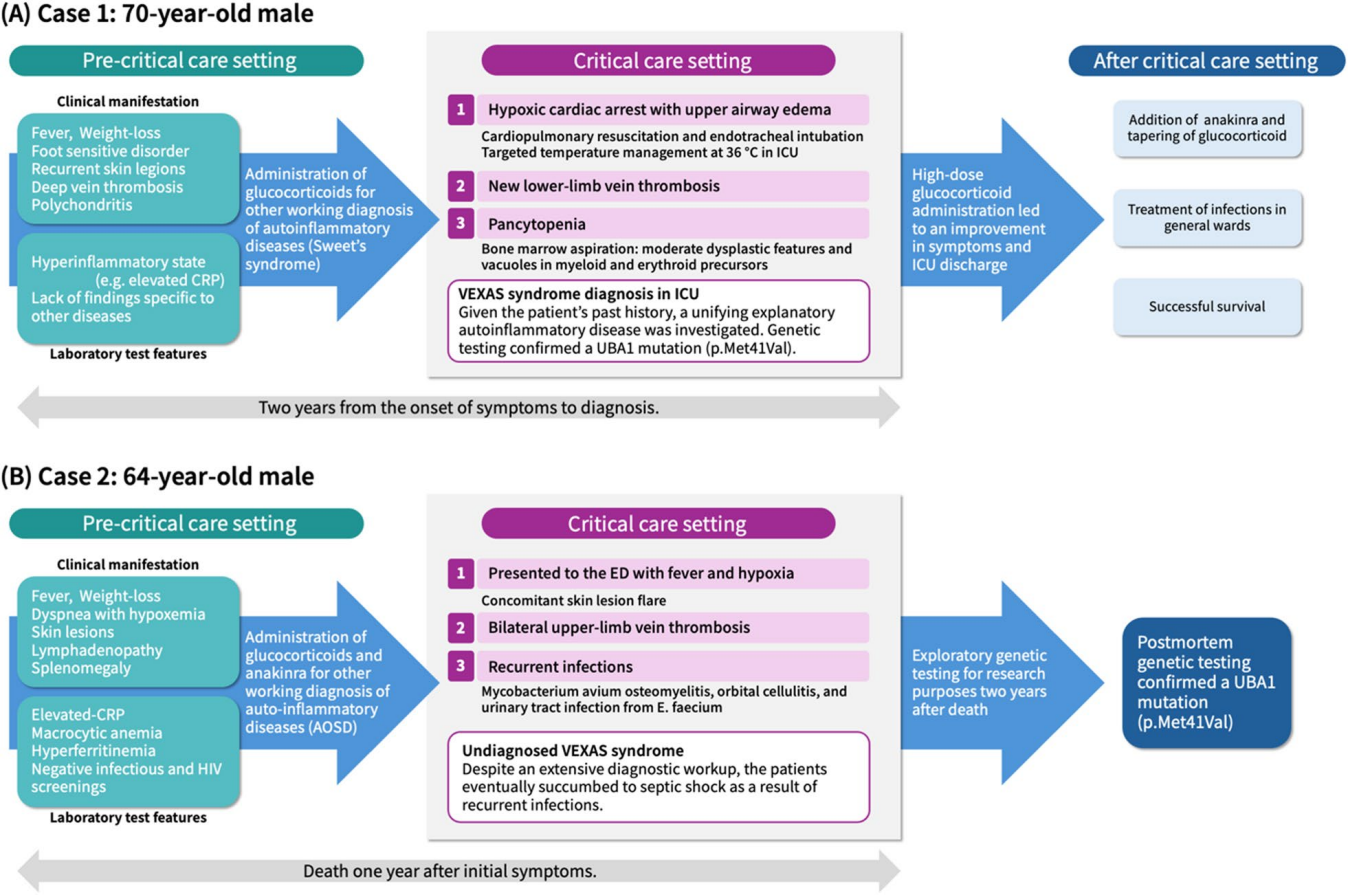
Clinical course charts of two model cases illustrating the trajectory from pre-diagnostic onset to critical care in VEXAS syndrome. Timelines were reconstructed from published case reports, highlighting systemic manifestations, diagnostic delay, and critical complications that ultimately led to definitive diagnosis. *VEXAS* Vacuoles, E1 enzyme, X-linked, Autoinflammatory, Somatic mutations, *CRP* C-reactive protein, *ICU* Intensive Care Unit, *ED* Emergency Department, *UBA1* Ubiquitin-like modifier activating enzyme 1, *AOSD* Adult-onset Still's disease

The identified trajectory is as follows: prior to definitive diagnosis, patients presented with a constellation of systemic manifestations, including persistent fever, weight loss, malaise, arthralgia, dyspnea, and cutaneous rashes, which are typical manifestations of VEXAS syndrome and often accompanied by concurrent inflammatory conditions such as chondritis, ophthalmitis, and thrombotic events. Laboratory studies revealed abnormalities: most notably, persistent elevation of inflammatory markers (C-reactive protein and erythrocyte sedimentation rate) accompanied by cytopenias ranging from isolated anemia to pancytopenia. Hyperferritinemia and hypergammaglobulinemia were also common, as were elevated cytokine profiles, particularly interleukin- 6. Radiologic findings showed pulmonary involvement in many cases. During the pre-diagnostic period, patients were often misclassified as rheumatic or autoinflammatory diseases, leading to the initiation of immunosuppressive or immunomodulatory therapy. While these therapeutic approaches occasionally contributed to symptom relief, they often led to severe complications, such as sepsis. The progression to critical states was marked by several life-threatening complications including upper airway inflammation leading to airway edema, acute kidney injury progressing to renal failure, HLH, diffuse alveolar hemorrhage, and shock. Figure 3 illustrates two cases in which undiagnosed VEXAS syndrome progressed to a critical state.

### Treatments and interventions in critical settings

Due to limited data on VEXAS syndrome in critical care settings, data on treatment approaches were primarily derived from case reports. Conventional supportive management was typically described, including antibiotic therapy and source control for infections [4, 5, 31, 40], mechanical ventilation for respiratory failure or altered consciousness [3, 14, 41], anticoagulation for thrombosis [41, 42], hemodialysis for renal dysfunction [13, 15], fluid resuscitation and vasopressor for shock [4, 19], and targeted temperature management following cardiac arrest [3].

When complicated by HLH, treatments typically included corticosteroids, immunosuppressants (anakinra, rituximab, ruxolitinib, sirolimus, siltuximab, cyclosporine), and chemotherapy (etoposide; CHOP regimen: cyclophosphamide, hydroxydaunorubicin, oncovin, prednisone) [12, 26, 34, 35]. Intravenous immunoglobulin and plasma exchange were also utilized [35]. Consequently, two of the four patients of HLH died.

Immunosuppressive and immunomodulatory treatments were also reported to address the marked inflammatory state of VEXAS syndrome. Corticosteroids were used for upper airway edema [22], cerebral sinus vein thrombosis [41], acute tubulointerstitial nephritis [13], alveolar hemorrhage[36], and distributive shock [19]. In certain cases, additional agents were combined with corticosteroids: azathioprine for encephalitis [14], anakinra for ARDS [17], and cyclosporine A, plasmapheresis, or tocilizumab for hyperinflammatory multi-organ failure [27].

## Discussion

To the best of our knowledge, this is the first scoping review to systematically analyze VEXAS syndrome in the critical care setting, providing insights on clinical characteristics and optimal treatment approaches on this recently identified syndrome. Although data on the clinical features and treatment approaches for VEXAS syndrome in the critical care setting were limited, our scoping review found that sepsis was a relatively common complication of VEXAS syndrome, and various types of shock, HLH, respiratory failure, thrombosis, and upper airway edema were also reported. Further, it is important to recognize that VEXAS syndrome can mimic sepsis and adult-onset Still's disease. Moreover, both the ICU admission rate (approximately 30%) and the mortality rate (ranging from 18 to 40%) were relatively high. In addition to standard critical care management, treatment approaches included immunosuppressive therapy and immunomodulatory therapy for symptoms caused by inflammation and exacerbation of VEXAS syndrome.

Our results highlight that a major challenge in understanding VEXAS syndrome in critically ill patients is the limited availability of comprehensive data. VEXAS syndrome is increasingly recognized as an important disease across multiple medical specialties, including hematology, rheumatology, ophthalmology, and dermatology. This syndrome is estimated to affect 1 in 4269 men over the age of 50 [1, 43] and may be more prevalent than currently expected. However, early recognition of VEXAS syndrome as an underlying disease in sepsis, shock, or respiratory failure remains difficult due to the limited awareness of VEXAS syndrome in the critical care field. Indeed, some cases of VEXAS syndrome included in this study were initially treated as sepsis or adult-onset Still's disease. Furthermore, it is likely that many cases of VEXAS syndrome remain undiagnosed and, consequently, unreported. In this context, our scoping review of VEXAS syndrome provides a potential diagnostic clue: patients presenting with unclear inflammatory syndromes—such as fever, weight loss, skin or lung involvement, chondritis, and thrombosis—may, over time, progress to severe inflammatory organ failure or sepsis under immunosuppression due to VEXAS syndrome, ultimately requiring critical care.

In the critical care setting, we found that managing VEXAS syndrome often requires not only standard critical care measures but also immunosuppressive and immunomodulatory therapy to control the inflammatory symptoms. However, our study revealed a lack of investigation into the specific immunosuppressive and immunomodulatory therapy regimens appropriate for different circumstances in the critical care setting. Moreover, our study suggests that sepsis is a common complication in patients with VEXAS syndrome undergoing immunosuppression or immunomodulation, posing a crucial challenge in patient care. Although immunomodulatory therapies have not traditionally been implemented in critical care, the coronavirus disease 2019 (COVID- 19) pandemic has increased awareness of targeted treatments, underscoring the need for critical care physicians to take an active role in this area [44]. Close collaboration with hematologists, rheumatologists, and other specialists is essential to optimize the management of VEXAS syndrome[45]. Furthermore, critical care physicians play a vital role as vigilant observers in identifying potential VEXAS syndrome cases among critically ill patients. As providers of life-sustaining interventions, critical care physicians collaborate with other specialists to manage complications of immunosuppressive therapy, ensuring comprehensive care for these complex cases.

The main limitation of this study is the insufficient data and publications on VEXAS syndrome in critical care settings, largely due to underdiagnosis and publication bias. As a result, case reports served as the leading source of information for this review. Additionally, due to resource constraints, including challenges in reliable translation, we limited our review to English-language publications, which may have introduced selection bias. Given the limited available literature, this review may not fully capture the spectrum of VEXAS syndrome presentation and management in critical care. Furthermore, our narrowly defined search terms, limited to confirmed VEXAS syndrome cases, may have overlooked misdiagnosed or unreported cases, potentially underestimating the true incidence and clinical diversity. Moreover, the subjective determination of report eligibility due to unclear severity criteria, may have introduced selection bias. While our study highlights the diverse clinical manifestations of VEXAS syndrome in critically ill patients, there remains a lack of systematic data to guide early recognition and management. Our findings underscore the need for future investigation in these unexplored areas. Specifically, a multicenter international retrospective or prospective cohort study or registry on VEXAS syndrome in the critical care setting would provide valuable insights into precise ICU admission rate and associated complications, as well as the efficacy and safety of optimal treatment. Such studies are essential for identifying the risk factors of critical illness in VEXAS syndrome and may ultimately contribute to the development of standardized treatment guidelines to improve patients outcomes.

## Conclusion

VEXAS syndrome presents with diverse clinical manifestations in the critical care setting and can lead to severe complications such as sepsis, shock, HLH, respiratory failure, and thrombosis. It is imperative for critical care providers to recognize and differentiate VEXAS syndrome from other diseases to ensure optimal treatment including conventional critical care management as well as immunosuppressive and immunomodulatory therapies. Our findings should facilitate further research and clinical attention to VEXAS syndrome in the critical care setting.

## Supplementary Information

Additional file 1.

## Data Availability

All data generated and analyzed during this study are included in this published article and its supplementary materials.

## Abbreviations

ARDS: Acute respiratory distress syndrome
CENTRAL: Cochrane Central Register of Controlled Trials
CHOP: Cyclophosphamide, hydroxydaunorubicin, oncovin, prednisone
COVID- 19: Coronavirus disease 2019
CRP: C-reactive protein
ED: Emergency Department
HLH: Hemophagocytic lymphohistiocytosis
ICU: Intensive Care Unit
JBI: Joanna Briggs Institute
PRISMA-ScR: Systematic Reviews and Meta-Analysis extension for Scoping Reviews guidelines
UBA1: Ubiquitin-like modifier activating enzyme 1
VEXAS: Vacuoles, E1 enzyme, X-linked, Autoinflammatory, Somatic mutations

## References

[1] Beck DB, Ferrada MA, Sikora KA, Ombrello AK, Collins JC, Pei W, et al. Somatic mutations in UBA1 and severe adult-onset autoinflammatory disease. N Engl J Med. 2020;383:2628–38. 33108101 10.1056/NEJMoa2026834 PMC7847551

[2] Al-Hakim A, Savic S. An update on VEXAS syndrome. Expert Rev Clin Immunol. 2023;19:203–15. 36537591 10.1080/1744666X.2023.2157262

[3] Belicard F, Belhomme N, Bouzy S, Saillard C, Nedelec F, Mear J-B, et al. Vacuoles, E1 enzyme, X-linked, autoinflammatory, and somatic syndrome in the intensive care unit: a case report. J Med Case Rep. 2023;17:314. 37480098 10.1186/s13256-023-04034-5 PMC10362754

[4] Wilson NR, Jain P, Gomez JA, Lu H, Pemmaraju N. Concurrent myelodysplasia and monoclonal B lymphocytosis in VEXAS syndrome. Leuk Res. 2022;120: 106909. 35820269 10.1016/j.leukres.2022.106909

[5] van der Made CI, Potjewijd J, Hoogstins A, Willems HPJ, Kwakernaak AJ, de Sevaux RGL, et al. Adult-onset autoinflammation caused by somatic mutations in UBA1: a Dutch case series of patients with VEXAS. J Allergy Clin Immunol. 2022;149:432-9.e4. 34048852 10.1016/j.jaci.2021.05.014

[6] Satoh K. Clinical features and management of VEXAS syndrome in critical care: a scoping review protocol v2 [Internet]. 2024. Available from: 10.17504/protocols.io.14egn621ql5d/v4

[7] Tricco AC, Lillie E, Zarin W, O’Brien KK, Colquhoun H, Levac D, et al. PRISMA extension for scoping reviews (PRISMA-ScR): checklist and explanation. Ann Intern Med. 2018;169:467–73. 30178033 10.7326/M18-0850

[8] Casal Moura M, Baqir M, Tandon YK, Samec MJ, Hines AS, Reichard KK, et al. Pulmonary manifestations in VEXAS syndrome. Respir Med. 2023;213: 107245. 37062498 10.1016/j.rmed.2023.107245

[9] Gurnari C, McLornan DP. Update on VEXAS and role of allogeneic bone marrow transplant: considerations on behalf of the Chronic Malignancies Working Party of the EBMT. Bone Marrow Transplant. 2022;57:1642–8. 35941354 10.1038/s41409-022-01774-8

[10] Ferrada MA, Sikora KA, Luo Y, Wells KV, Patel B, Groarke EM, et al. Somatic mutations in UBA1 define a distinct subset of relapsing polychondritis patients with VEXAS. Arthritis Rheumatol. 2021;73:1886–95. 33779074 10.1002/art.41743 PMC13539413

[11] de Valence B, Delaune M, Nguyen Y, Jachiet V, Heiblig M, Jean A, et al. Serious infections in patients with VEXAS syndrome: data from the French VEXAS registry. Ann Rheum Dis. 2024;83:372–81. 38071510 10.1136/ard-2023-224819

[12] Grey A, Cheong PL, Lee FJ, Abadir E, Favaloro J, Yang S, et al. A case of VEXAS syndrome complicated by hemophagocytic lymphohistiocytosis. J Clin Immunol. 2021;41:1648–51. 34080084 10.1007/s10875-021-01070-y

[13] Ronsin C, Benard L, Mourtada A, Perrin F, Boukerroucha Z. Acute tubulointerstitial nephritis revealing VEXAS syndrome. Kidney Int. 2022;101:1295–7. 35597594 10.1016/j.kint.2022.03.012

[14] Johnsson M. Rhombencephalitis in a patient with VEXAS syndrome. Neuroimmunol Rep. 2023;4: 100176.

[15] Sardarli K, Del Bosque A, Yao M, Cunard R, Miracle C. 10 VEXAS, a vexing clinical case with renal involvement. Am J Kidney Dis. 2024;83:S3-4.

[16] Rempenault C, Lukas C, Combe B, Herrero A, Pane I, Schaeverbeke T, et al. Risk of diverticulitis and gastrointestinal perforation in rheumatoid arthritis treated with tocilizumab compared to rituximab or abatacept. Rheumatology (Oxford). 2022;61:953–62. 33993216 10.1093/rheumatology/keab438

[17] van Leeuwen-Kerkhoff N, de Witte MA, Heijstek MW, Leavis HL. Case report: up-front allogeneic stem cell transplantation in a patient with the VEXAS syndrome. Br J Haematol. 2022;199:e12–5. 36039520 10.1111/bjh.18424

[18] Grambow-Velilla J, Braun T, Pop G, Louzoun A, Soussan M. Aortitis PET imaging in VEXAS syndrome: a case report. Clin Nucl Med. 2023;48:e67–8. 36607374 10.1097/RLU.0000000000004506

[19] Ciprian G. Adverse reaction to COVID-19 mRNA vaccination in a patient with VEXAS syndrome. Cureus. 2022;14: e23456. 35481304 10.7759/cureus.23456 PMC9034849

[20] Georgin-Lavialle S, Terrier B, Guedon AF, Heiblig M, Comont T, Lazaro E, et al. Further characterization of clinical and laboratory features in VEXAS syndrome: large-scale analysis of a multicentre case series of 116 French patients. Br J Dermatol. 2022;186:564–74. 34632574 10.1111/bjd.20805

[21] Groarke EM, Dulau-Florea AE, Kanthi Y. Thrombotic manifestations of VEXAS syndrome. Semin Hematol. 2021;58:230–8. 34802545 10.1053/j.seminhematol.2021.10.006

[22] Guerrero-Bermúdez CA, Cardona-Cardona AF, Ariza-Parra EJ, Arostegui JI, Mensa-Vilaro A, Yague J, et al. Vacuoles, E1 enzyme, X-linked, autoinflammatory, somatic syndrome (VEXAS syndrome) with prominent supraglottic larynx involvement: a case-based review. Clin Rheumatol. 2022;41:3565–72. 35986821 10.1007/s10067-022-06338-1

[23] Muratore F, Marvisi C, Castrignanò P, Nicoli D, Farnetti E, Bonanno O, et al. VEXAS syndrome: a case series from a single-center cohort of Italian patients with vasculitis. Arthritis Rheumatol. 2022;74:665–70. 34611997 10.1002/art.41992 PMC8957507

[24] Myint K, Patrao N, Vonica O, Vahdani K. Recurrent superior orbital fissure syndrome associated with VEXAS syndrome. J Ophthalmic Inflamm Infect. 2023;13:39. 37673972 10.1186/s12348-023-00362-1 PMC10482812

[25] Yamaguchi H, Kobayashi D, Nakamura G, Aida R, Horii Y, Okamoto T, et al. Acute heart failure due to left common iliac arteriovenous fistula: a case of VEXAS syndrome. Mod Rheumatol Case Rep. 2023;7:327–33. 36264203 10.1093/mrcr/rxac082

[26] Staels F, Betrains A, Woei-A-Jin FJSH, Boeckx N, Beckers M, Bervoets A, et al. Case report: VEXAS syndrome: from mild symptoms to life-threatening macrophage activation syndrome. Front Immunol. 2021;12: 678927. 34046042 10.3389/fimmu.2021.678927 PMC8147557

[27] Matsumoto H, Asano T, Tsuchida N, Maeda A, Yoshida S, Yokose K, et al. Behçet’s disease with a somatic UBA1 variant: expanding spectrum of autoinflammatory phenotypes of VEXAS syndrome. Clin Immunol. 2022;238: 108996. 35398520 10.1016/j.clim.2022.108996

[28] Więsik-Szewczyk E, Zegadło A, Sobczyńska-Tomaszewska A, Korzeniowska M, Jahnz-Rózyk K. Case report: VEXAS as an example of autoinflammatory syndrome in pulmonology clinical practice. Front Med (Lausanne). 2024;11:1340888. 38343641 10.3389/fmed.2024.1340888 PMC10858452

[29] Austestad J, Madland TM, Sandnes M, Haslerud TM, Benneche A, Reikvam H. VEXAS syndrome in a patient with myeloproliferative neoplasia. Case Rep Hematol. 2023;2023:6551544. 36879894 10.1155/2023/6551544 PMC9985496

[30] Riescher S, Lecomte R, Danic G, Graveleau J, Le Bris Y, Hello M, et al. Susceptibility to mycobacterial infection in VEXAS syndrome. Rheumatology (Oxford). 2024. 10.1093/rheumatology/keae087. 10.1093/rheumatology/keae087 38317027

[31] Shimizu T, Ide H, Tsuji Y, Koga T, Kawakami A. VEXAS syndrome complicated with severe infection. Rheumatology (Oxford). 2022;61:e374–6. 35723601 10.1093/rheumatology/keac364

[32] Wilke MVMB, Morava-Kozicz E, Koster MJ, Schmitz CT, Foster SK, Patnaik M, et al. Exome sequencing can misread high variant allele fraction of somatic variants in UBA1 as hemizygous in VEXAS syndrome: a case report. BMC Rheumatol. 2022;6:54. 36038944 10.1186/s41927-022-00281-z PMC9426024

[33] Islam S, Cullen T, Sumpton D, Damodaran A, Heath D, Bosco A, et al. VEXAS syndrome: lessons learnt from an early Australian case series. Intern Med J. 2022;52:658–62. 35419965 10.1111/imj.15742

[34] Kao RL, Jacobsen AA, Billington CJ Jr, Yohe SL, Beckman AK, Vercellotti GM, et al. A case of VEXAS syndrome associated with EBV-associated hemophagocytic lymphohistiocytosis. Blood Cells Mol Dis. 2022;93: 102636. 34864445 10.1016/j.bcmd.2021.102636

[35] Miyoshi Y, Kise T, Morita K, Okada H, Imadome K-I, Tsuchida N, et al. Long-term remission of VEXAS syndrome achieved by a single course of CHOP therapy: a case report. Mod Rheumatol Case Rep. 2023;8:199–204. 37548220 10.1093/mrcr/rxad041

[36] Sofi FA, Naqati SM, Ahmad M, Bindroo M. VEXAS syndrome presenting as diffuse alveolar haemorrhage. BMJ Case Rep. 2024;17: e259474. 38538102 10.1136/bcr-2023-259474 PMC10982693

[37] Suárez-Díaz S, Yllera-Gutiérrez C, Morán-Castaño C, Caminal-Montero L. Entities inside one another: VEXAS, a matryoshka-type disease. Reumatol Clín (Engl Ed). 2024;20:57–8. 38233010 10.1016/j.reumae.2023.08.002

[38] Sharma A, Naidu G, Deo P, Beck DB. VEXAS syndrome with systemic lupus erythematosus: expanding the spectrum of associated conditions. Arthritis Rheumatol. 2022;74:369–71. 34463053 10.1002/art.41957 PMC8795469

[39] Varadarajan A, Verghese RM, Tirlangi PK, Dass J, Soneja M, Seth T. VEXAS syndrome (vacuoles, E1 enzyme, X-linked, autoinflammatory, somatic). QJM. 2023;116:313–5. 36409014 10.1093/qjmed/hcac259

[40] Salehi T, Callisto A, Beecher MB, Hissaria P. Tofacitinib as a biologic response modifier in VEXAS syndrome: a case series. Int J Rheum Dis. 2023;26:2340–3. 37337622 10.1111/1756-185X.14785

[41] Zisapel M, Seyman E, Molad J, Hallevi H, Mauda-Havakuk M, Jonas-Kimchi T, et al. Case report: cerebral sinus vein thrombosis in VEXAS syndrome. Front Med (Lausanne). 2024;11:1377768. 38651063 10.3389/fmed.2024.1377768 PMC11033418

[42] Abumanhal M, Leibovitch I, Zisapel M, Eviatar T, Edel Y, Ben CR. Ocular and orbital manifestations in VEXAS syndrome. Eye. 2024;38:1748–54. 38548942 10.1038/s41433-024-03014-3 PMC11156927

[43] Ruffer N, Krusche M. VEXAS syndrome: a diagnostic puzzle. RMD Open. 2023. 10.1136/rmdopen-2023-003332. 37532466 10.1136/rmdopen-2023-003332 PMC10401208

[44] Janssen M, Endeman H, Bos LDJ. Targeted immunomodulation: a primer for intensivists. Intensive Care Med. 2023;49:462–4. 36867231 10.1007/s00134-023-07009-8 PMC9982766

[45] Bica BERG, de Souza AWS, Pereira IA. Unveiling the clinical spectrum of relapsing polychondritis: insights into its pathogenesis, novel monogenic causes, and therapeutic strategies. Adv Rheumatol. 2024;64:29. 38627861 10.1186/s42358-024-00365-z PMC13488845

[46] Borie R, Debray MP, Guedon AF, Mekinian A, Terriou L, Lacombe V, et al. Pleuropulmonary manifestations of vacuoles, E1 enzyme, X-linked, autoinflammatory, somatic (VEXAS) syndrome. Chest. 2023;163:575–85. 36272567 10.1016/j.chest.2022.10.011

[47] Comont T, Heiblig M, Rivière E, Terriou L, Rossignol J, Bouscary D, et al. Azacitidine for patients with Vacuoles, E1 Enzyme, X-linked, Autoinflammatory, Somatic syndrome (VEXAS) and myelodysplastic syndrome: data from the French VEXAS registry. Br J Haematol. 2022;196:969–74. 34651299 10.1111/bjh.17893

[48] De Valence de Minardière B, Delaune M, Nguyen Y, Jachiet V, Heiblig M, Jean A, et al. Serious infections in patients with vexas syndrome: a study from the French vexas group. Blood. 2023;142:5599–5599.

[49] Comont T, Kosmider O, Heiblig M, Terrier B, Bouscary D, Le Guenno G, et al. Azacitidine for patients with vexas syndrome: data from the French vexas registry. Blood. 2023;142:4604.

[50] Kusne Y, Koster M, Warrington K, Go R, Lasho T, Finke C, et al. Monocytopenia as an adverse prognosticator in vexas syndrome. Blood. 2022;140:11406–7.

[51] Gurnari C, Pascale MR, Vitale A, Diral E, Tomelleri A, Galossi E, et al. Diagnostic capabilities, clinical features, and longitudinal UBA1 clonal dynamics of a nationwide VEXAS cohort. Am J Hematol. 2024;99:254–62. 38108611 10.1002/ajh.27169

[52] Alcedo Andrade PE, Shalhoub R, Dulau-Florea A, Nghiem K, Ferrada M, Wilson L, et al. Thrombotic manifestations in patients with vexas syndrome. Blood. 2022;140:2788–9.35981475

[53] Heiblig M, Ferrada MA, Koster MJ, Barba T, Gerfaud-Valentin M, Mékinian A, et al. Ruxolitinib is more effective than other JAK inhibitors to treat VEXAS syndrome: a retrospective multicenter study. Blood. 2022;140:927–31. 35609174 10.1182/blood.2022016642 PMC9412002

[54] Gutierrez-Rodrigues F, Kusne Y, Fernandez J, Lasho T, Shalhoub R, Ma X, et al. Spectrum of clonal hematopoiesis in VEXAS syndrome. Blood. 2023;142:244–59. 37084382 10.1182/blood.2022018774 PMC10375269

[55] Kusne Y, Ghorbanzadeh A, Dulau-Florea A, Shalhoub R, Alcedo PE, Nghiem K, et al. Venous and arterial thrombosis in patients with VEXAS syndrome. Blood. 2024;143:2190–200. 38306657 10.1182/blood.2023022329 PMC11143532

[56] Vitale A, Caggiano V, Martin-Nares E, Frassi M, Dagna L, Hissaria P, et al. Orbital/ocular inflammatory involvement in VEXAS syndrome: data from the international AIDA network VEXAS registry. Semin Arthritis Rheum. 2024;66: 152430. 38554594 10.1016/j.semarthrit.2024.152430

[57] Heiblig M, Ferrada MA, Gerfaud-Valentin M, Barba T, Mékinian A, Koster M, et al. Clinical efficacy of JAK inhibitors in patients with vexas syndrome: a multicenter retrospective study. Blood. 2021;138:2608–2608.

