# Supplementary material for: Clinical features and treatments of VEXAS syndrome in critical care: a scoping review
Source: Crit Care. 2025 Apr 17;29:154. doi: 10.1186/s13054-025-05390-y (PMC12004820; doi:10.1186/s13054-025-05390-y)
Supplement: Supplementary file 1 — Additional file 1. [file 13054_2025_5390_MOESM1_ESM.docx]

**Supplementary section: Difference between protocol and review**

In our protocol, we initially planned to use a standardized data collection spreadsheet for extraction by two independent reviewers. However, due to the unexpectedly large volume of articles that required full-text screening, we modified our approach to maintain efficiency while preserving methodological rigor.

Instead of direct extraction into spreadsheets, our two clinically experienced reviewers (KS and KO) independently highlighted relevant data points within shared PDF documents. One reviewer then compiled these independently marked data into a final spreadsheet, after which both reviewers verified the accuracy and reached consensus on the final dataset. This approach allowed us to process the large volume of literature while maintaining independent assessment and final consensus verification.

**Supplementary Table 1.** Search strategies for the study selection of each database

**Abbreviation:** VEXAS, Vacuoles, E1 enzyme, X-linked, Autoinflammatory, Somatic mutations

| PubMed search strategy | “VEXAS”[tiab] OR “Vacuoles, E1 enzyme, X-linked, Autoinflammatory, Somatic”[tiab] |
| --- | --- |
| CENTRAL search strategy | “VEXAS”:ti,ab OR “Vacuoles, E1 enzyme, X-linked, Autoinflammatory, Somatic”:ti,ab |
| Embase search strategy | ti(VEXAS) OR ab(VEXAS) OR ti(Vacuoles, E1 enzyme, X-linked, Autoinflammatory, Somatic) OR ab(Vacuoles, E1 enzyme, X-linked, Autoinflammatory, Somatic) |
| Web of Science search strategy | ​​"VEXAS" OR "Vacuoles, E1 enzyme, X-linked, Autoinflammatory, Somatic" |

**Supplementary Table 2.** List of excluded articles and reasons for exclusion after full-text screening

| **Reason for exclusion** | **Excluded articles** |
| --- | --- |
| Wrong publication type *e.g.* comment (n = 3) | 1. Clonal hematopoiesis in VEXAS syndrome. Blood. 2023;142(3):306. 2. Rogez J, Ravaiau C, Lacombe V. Comment on: Efficient detection of somatic UBA1 variants and clinical scoring system predicting patients with variants in VEXAS syndrome. Rheumatology. 2024;63(8):e231-3. 3. Tsuchida N, Uchiyama Y, Maeda A, Horita N, Kirino Y, Matsumoto N. Comment on: Efficient detection of somatic UBA1 variants and clinical scoring system predicting patients with variants in VEXAS syndrome: reply. Rheumatology. 2024;63(8):e229-e230. |
| Wrong population *i.e.* not related to critical care (n = 271) | 1. Nguyen JK, Lee A. Novel VEXAS (Vacuoles, E1 enzyme, X-linked, autoinflammatory, somatic) syndrome identified in a patient presenting with Sweet's Syndrome . Australas J Dermatol. 2022;63:28-32. 2. Gurnari C, McLornan DP. Update on VEXAS and role of allogeneic bone marrow transplant: Considerations on behalf of the Chronic Malignancies Working Party of the EBMT. Bone Marrow Transplant. 2022;57:1642–8. 3. Koster MJ, Kourelis T, Reichard KK, Kermani TA, Beck DB, Cardona DO, et al. Clinical heterogeneity of the VEXAS syndrome: A case series. Mayo Clin Proc. 2021;96:2653–9. 4. Mohammed TO, Alavi A, Aghazadeh N, Koster MJ, Olteanu H, Mangaonkar AA, et al. Vacuoles, E1 enzyme, X-linked, autoinflammatory, somatic (VEXAS) syndrome: a presentation of two cases with dermatologic findings. Int J Dermatol. 2023;62:e313–5. 5. Tozaki N, Tawada C, Niwa H, Mizutani Y, Shu E, Kawase A, et al. A case of VEXAS syndrome (vacuoles, E1 enzyme, X-linked, autoinflammatory, somatic) with decreased oxidative stress levels after oral prednisone and tocilizumab treatment. Front Med (Lausanne). 2022;9:1046820. 6. Grayson PC, Patel BA, Young NS. VEXAS syndrome. Blood. 2021;137:3591–4. 7. Koster MJ, Lasho TL, Olteanu H, Reichard KK, Mangaonkar A, Warrington KJ, et al. VEXAS syndrome: Clinical, hematologic features and a practical approach to diagnosis and management. Am J Hematol. 2024;99:284–99. 8. Lokhande A, Jarmale S, Vaishnav YJ, Schaefer J. An orbital manifestation of VEXAS syndrome. Ophthal Plast Reconstr Surg. 2023;39:e194–7. 9. Khitri M-Y, Guedon AF, Georgin-Lavialle S, Terrier B, Saadoun D, Seguier J, et al. Comparison between idiopathic and VEXAS-relapsing polychondritis: analysis of a French case series of 95 patients. RMD Open. 2022;8:e002255. 10. Bica BERG, de Souza AWS, Pereira IA. Unveiling the clinical spectrum of relapsing polychondritis: insights into its pathogenesis, novel monogenic causes, and therapeutic strategies. Adv Rheumatol. 2024;64:29. 11. Hines AS, Koster MJ, Bock AR, Go RS, Warrington KJ, Olteanu H, et al. Targeted testing of bone marrow specimens with cytoplasmic vacuolization to identify previously undiagnosed cases of VEXAS syndrome. Rheumatology (Oxford). 2023;62:3947–51. 12. Goyal A, Narayanan D, Wong W, Laga AC, Connell NT, Ritter SY, et al. Tocilizumab for treatment of cutaneous and systemic manifestations of vacuoles, E1 enzyme, X-linked, autoinflammatory, somatic (VEXAS) syndrome without myelodysplastic syndrome. JAAD Case Rep. 2022;23:15–9. 13. Campochiaro C, Tomelleri A, Cavalli G, De Luca G, Grassini G, Cangi MG, et al. Successful use of cyclosporin A and interleukin-1 blocker combination therapy in VEXAS syndrome: a single-center case series. Arthritis Rheumatol. 2022;74:1302–3. 14. Philip R, Cadro V, Aouba A, Chantepie S, Bracquemart C, Dumont A. VEXAS syndrome: A new mimicker of idiopathic multicentric Castleman disease. Joint Bone Spine. 2024;91:105731. 15. Templé M, Kosmider O. VEXAS syndrome: A novelty in MDS landscape. Diagnostics (Basel). 2022;12:1590. 16. Fukuda N, Kanai D, Hoshino K, Fukuda Y, Morita R, Ishikawa Y, et al. Vacuoles, E1 enzyme, X-linked, autoinflammatory, somatic (VEXAS) syndrome presenting as recurrent aseptic peritonitis in a patient receiving peritoneal dialysis: a case report. BMC Nephrol. 2024;25:18. 17. Al-Hakim A, Savic S. An update on VEXAS syndrome. Expert Rev Clin Immunol. 2023;19:203–15. 18. Kreutzinger V, Pankow A, Boyadzhieva Z, Schneider U, Ziegeler K, Stephan LU, et al. VEXAS and myelodysplastic syndrome: An interdisciplinary challenge. J Clin Med. 2024;13:1049. 19. Vivekanantham A, Kesavan M, Evans J, Matin RN, Elliott I, Luqmani R. Pos1453 overlapping features of relapsing polychondritis and sweet’s syndrome: Could this be vexas (vacuoles, ubiquitin a1e mutation, x-linked, autoinflammatory, somatic) syndrome? Ann Rheum Dis. 2021;80:1010–1. 20. Kouranloo K, Ashley A, Zhao S, Dey M. Pulmonary manifestations in VEXAS (Vacuoles, E1 enzyme, X-linked, autoinflammatory, somatic) syndrome: A systematic literature review. Arthritis Rheumatol. 2022;74(Suppl 9):1634-5 21. Diaz MJ, Liu VY, Motaparthi K. Noninflammatory extrafacial edema as a clue to the diagnosis of vacuoles, E1 enzyme, X-linked, autoinflammatory, somatic (VEXAS) syndrome. JAAD Case Rep. 2024;47:96–8. 22. Takahashi N, Takeichi T, Nishida T, Sato J, Takahashi Y, Yamamura M, et al. Extensive multiple organ involvement in VEXAS syndrome. Arthritis Rheumatol. 2021;73:1896–7. 23. Stubbins RJ, Cherniawsky H, Chen LYC, Nevill TJ. Innovations in genomics for undiagnosed diseases: vacuoles, E1 enzyme, X-linked, autoinflammatory, somatic (VEXAS) syndrome. CMAJ. 2022;194:E524–7. 24. Sujobert P, Heiblig M, Jamilloux Y. VEXAS: where do we stand 2 years later? Curr Opin Hematol. 2023;30:64–9. 25. Afsahi V, Christensen RE, Alam M. VEXAS syndrome in dermatology. Arch Derm Res. 2023;315:161–4. 26. Alhomida F, Beck DB, George TI, Shaffer A, Lebiedz-Odrobina D, Kovacsovics T, et al. Vacuoles, E1 enzyme, X-linked, autoinflammatory, somatic (VEXAS) syndrome-clinical presentation of a newly described somatic, autoinflammatory syndrome. JAAD Case Rep. 2021;14:111–3. 27. Vitale A, Caggiano V, Della Casa F, Hernández-Rodríguez J, Frassi M, Monti S, et al. Development and implementation of the AIDA international registry for patients with VEXAS syndrome. Front Med (Lausanne). 2022;9:926500. 28. Podvin B, Cleenewerck N, Nibourel O, Marceau-Renaut A, Roynard P, Preudhomme C, et al. Three UBA1 clones for a unique VEXAS syndrome. Rheumatology (Oxford). 2024;63:e48–50. 29. Grambow-Velilla J, Braun T, Pop G, Louzoun A, Soussan M. Aortitis PET imaging in VEXAS syndrome: A case report. Clin Nucl Med. 2023;48:e67–8. 30. Sloan B. This month in JAAD Case Reports: November 2021. Vacuoles, E1 enzyme, X-linked, autoinflammatory, somatic (VEXAS) syndrome-A newly described autoinflammatory disease. J Am Acad Dermatol. 2021;85:1111. 31. Wang CX, Yokoyama CC, Rosman IS, Musiek AC. Extensive reactive cutaneous histiocytic infiltrate resembling non-Langerhans cell histiocytosis as the presenting sign of underlying vacuoles, E1 enzyme, X-linked, autoinflammatory, somatic syndrome. JAAD Case Rep. 2024;43:20–3. 32. Argobi Y. VEXAS syndrome with cutaneous nodules. Dermatol Reports. 2022;14:9414. 33. Padureanu V, Marinaș CM, Bobirca A, Padureanu R, Patrascu S, Dascalu AM, et al. Clinical manifestations in vacuoles, E1 enzyme, X-linked, autoinflammatory, somatic (VEXAS) syndrome: A narrative review. Cureus. 2024;16:e53041. 34. Dehghan N, Marcon KM, Sedlic T, Beck DB, Dutz JP, Chen LYC. Vacuoles, E1 enzyme, X-linked, autoinflammatory, somatic (VEXAS) syndrome: fevers, myalgia, arthralgia, auricular chondritis, and erythema nodosum. Lancet. 2021;398:621. 35. Ang T, Tong JY, Selva D. Orbital inflammatory disease due to VEXAS syndrome: An entity to consider. Ophthal Plast Reconstr Surg. 2024;40:116–7. 36. Stubbins RJ. Lost in translation: cytoplasmic UBA1 and VEXAS syndrome. Blood. 2022;140:1455–7. 37. Williams S, Stewart M, Fifi-Mah A, Dehghan N. VEXAS syndrome: Clinical case series from a Canadian cohort. J Rheumatol. 2024;51:734–7. 38. Fagart A, Quemeneur T, Collet G, Demailly F, Rousselin C. A “leopard man” aspect on 18 F-FDG PET/CT revealing a VEXAS syndrome. Clin Nucl Med. 2023;48:e33–4. 39. Strasser B, Kranewitter W, Hofer H, Haushofer A. Bone marrow reinvestigation leading to the diagnosis of VEXAS syndrome. Lab Med. 2024;55:655–7. 40. Yildirim F, Erdogan M, Yalcin Mutlu M, Akkuzu G, Ozgur DS, Karaalioglu B, et al. VEXAS syndrome with severe multisystem involvement: Rapid recovery after splenectomy. Int J Rheum Dis. 2023;26:559–62. 41. Sujobert P, Largeaud L, Jamilloux Y, Heiblig M, Kosmider O. VEXAS: is it time to reshape the nosology of clonal hematopoiesis? Expert Rev Hematol. 2023;16:495–9. 42. Ruffer N, Krusche M. VEXAS syndrome: a diagnostic puzzle. RMD Open 2023;9:e003332. 43. Loschi M, Roux C, Sudaka I, Ferrero-Vacher C, Marceau-Renaut A, Duployez N, et al. Allogeneic stem cell transplantation as a curative therapeutic approach for VEXAS syndrome: a case report. Bone Marrow Transplant. 2022;57:315–8. 44. Huang H, Zhang W, Cai W, Liu J, Wang H, Qin T, et al. VEXAS syndrome in myelodysplastic syndrome with autoimmune disorder. Exp Hematol Oncol. 2021;10:23. 45. Lacombe V, Hadjadj J, Georgin-Lavialle S, Lavigne C, Geneviève F, Kosmider O. Vacuoles in bone marrow progenitors: VEXAS syndrome and beyond. Lancet Haematol. 2024;11:e160–7. 46. Watanabe R, Kiji M, Hashimoto M. Vasculitis associated with VEXAS syndrome: A literature review. Front Med (Lausanne). 2022;9:983939. 47. Ding Y, Dulau-Florea AE, Groarke EM, Patel BA, Beck DB, Grayson PC, et al. Use of flow cytometric light scattering to recognize the characteristic vacuolated marrow cells in VEXAS syndrome. Blood Adv. 2023;7:6151–5. 48. Pathmanathan K, Taylor E, Balendra J, Lim A, Carroll G. VEXAS syndrome: favourable clinical and partial haematological responses to subcutaneous abatacept therapy with 30-month follow-up. Rheumatology (Oxford). 2022;61:e174–7. 49. Oganesyan A, Hakobyan Y, Terrier B, Georgin-Lavialle S, Mekinian A. Looking beyond VEXAS: Coexistence of undifferentiated systemic autoinflammatory disease and myelodysplastic syndrome. Semin Hematol. 2021;58:247–53. 50. Lötscher F, Seitz L, Sarbu A, Maurer B. Genetic double strike: VEXAS and TET2 positive myelodysplastic syndrome in a patient with long-standing refractory autoinflammatory disease. Case report. Swiss Med Wkly. 151(Suppl 252):5S. 51. Kirino Y. Clinical challenges of emerging acquired autoinflammatory diseases, including VEXAS syndrome. Intern Med. 2025;64:25–30. 52. Bellman P, Gonzalez-Lugo JD, Shahzad M, Amin MK, Khalid MF, Suleman N, et al. Successful treatment with fludarabine and cyclophosphamide in a VEXAS syndrome patient with associated myelodysplastic syndrome: a case report and systematic review. Front Oncol. 2024;14:1383730. 53. Mascaro JM, Rodriguez-Pinto I, Poza G, Mensa-Vilaro A, Fernandez-Martin J, Caminal-Montero L, et al. Spanish cohort of VEXAS syndrome: clinical manifestations, outcome of treatments and novel evidences about UBA1 mosaicism. Ann Rheum Dis. 2023;82:1594–605. 54. Patel BA, Young NS. Towards treatments for VEXAS. Br J Haematol. 2022;196:804–5. 55. Lötscher F, Seitz L, Simeunovic H, Sarbu A-C, Porret NA, Feldmeyer L, et al. Case report: Genetic double strike: VEXAS and TET2-positive myelodysplastic syndrome in a patient with long-standing refractory autoinflammatory disease. Front Immunol. 2021;12:800149. 56. Chabrun F, Lacombe V, Dieu X, Geneviève F, Urbanski G. Accurate stratification between VEXAS syndrome and differential diagnoses by deep learning analysis of peripheral blood smears. Clin Chem Lab Med. 2023;61:1275–9. 57. Conway R. Ruxolitinib takes center stage for VEXAS syndrome. Blood. 2022;140:807–8. 58. Swiss Society Of Rheumatology. Supplementum 271: Abstracts of the Annual meeting of the Swiss Society of Rheumatology. Swiss Med Wkly. 2023;153:40135. 59. 32nd annual conference of the Australasian society of clinical immunology and allergy (ASCIA), 30 august - 2 September 2022. Intern Med J. 2022;52 Suppl 5:5–32. 60. Mischel A, Henry P, Hohnholt T, Sykes A, Moinizandi T. VEXAS syndrome: causing a fluid wave. J Hosp Med. 2023;18(Suppl 1):S6589. 61. Matsubara A, Tsuchida N, Sakurai M, Maeda A, Uchiyama Y, Sasaki K, et al. A case of VEXAS syndrome with Sweet’s disease and pulmonary involvement. J Dermatol. 2022;49:e177–8. 62. Duncan M, Basu T, Salisbury J, Kulasekararaj A, Sterling D. VEXAS syndrome: a case series. Br J Dermatol. 2022;187:91. 63. Kunimoto H, Miura A, Maeda A, Tsuchida N, Uchiyama Y, Kunishita Y, et al. Clinical and genetic features of Japanese cases of MDS associated with VEXAS syndrome. Int J Hematol. 2023;118:494–502. 64. Kataoka A, Mizumoto C, Kanda J, Iwasaki M, Sakurada M, Oka T, et al. Successful azacitidine therapy for myelodysplastic syndrome associated with VEXAS syndrome. Int J Hematol. 2023;117:919–24. 65. Groarke EM, Dulau-Florea AE, Kanthi Y. Thrombotic manifestations of VEXAS syndrome. Semin Hematol. 2021;58:230–8. 66. Terrier B, Posseme C, Temple M, Corneau A, Carbone F, Duroyon E, et al. VEXAS syndrome is characterized by blood and tissues inflammasome pathway activation and monocyte dysregulation [abstract]. Arthritis Rheumatol. 2022;74(Suppl 9). 67. Kosmider O, Possémé C, Templé M, Corneau A, Carbone F, Duroyon E, et al. VEXAS syndrome is characterized by inflammasome activation and monocyte dysregulation. Nat Commun. 2024;15:910. 68. Comont T, Heiblig M, Dion J, Riviere E, Terriou L, Rossignol J, et al. Azacitidine (AZA) for patients with vexas and myelodysplastic syndrome (MDS): Data from the French vexas registry. Blood. 2021;138:3689. 69. Maeda A, Tsuchida N, Uchiyama Y, Horita N, Kobayashi S, Kishimoto M, et al. Efficient detection of somatic UBA1 variants and clinical scoring system predicting patients with variants in VEXAS syndrome. Rheumatology (Oxford). 2024;63:2056–64. 70. Naidoo K, Venkatesan S, McGregor A, Fatah S. An elderly man with a relapsing and remitting neutrophilic dermatosis and final unifying diagnosis of VEXAS syndrome. Br J Dermatol. 2022;187(Suppl 1):144–5. 71. Obiorah IE, Patel BA, Groarke EM, Wang W, Trick M, Ombrello AK, et al. Benign and malignant hematologic manifestations in patients with VEXAS syndrome due to somatic mutations in UBA1. Blood Adv. 2021;5:3203–15. 72. Obiorah IE, Beck DB, Wang W, Ombrello A, Ferrada MA, Wu Z, et al. Myelodysplasia and bone marrow manifestations of somatic UBA1 mutated autoinflammatory disease. Blood. 2020;136:20–1. 73. Das S, Meena L. Unlocking the enigma: a case of POEMS syndrome (polyneuropathy, organomegaly, endocrinopathy, monoclonal protein, skin manifestation). Indian J Hematol Blood Transfus. 2023;39(Suppl 1):S21. 74. Shaukat F, Hart M, Burns T, Bansal P. UBA1 and DNMT3A mutations in VEXAS syndrome. A case report and literature review. Mod Rheumatol Case Rep. 2022;6:134–9. 75. Yildirim D, Inan MA, Akyurek N, Tufan A. VEXAS syndrome presenting as long COVID-19 [abstract]. Aktuelle Rheumatol. 2023;48(3):212–5. 76. Patel BA, Gutierrez-Rodrigues F, Kusne Y, Fernandez JA, Lasho T, Shalhoub R, et al. Clonal hematopoiesis in vexas syndrome. Blood. 2022;140:5745–6. 77. Matsuki Y, Kawai R, Suyama T, Katagiri K, Kanazawa N, Inaba Y. A case of VEXAS syndrome with myositis possibly associated with macrophage activation syndrome. J Dermatol. 2022;49:e441–3. 78. Ospina Cardona D, Wilson LL, Ferrada MA, Ombrello AK, Grayson PC, Aksentijevich I, et al. Defining clinical and genetic hallmarks of VEXAS syndrome [abstract]. Pediatr Rheumatol. 2022;20(Suppl 1):2. 79. Mangaonkar A, Langer KJ, Lasho T, Finke C, Litzow MR, Hogan WJ, et al. Pilot prospective study of reduced intensity conditioning allogeneic hematopoietic stem cell transplantation in patients with vexas syndrome. Transplant Cell Ther. 2024;30:S292. 80. Sajawal S, Green M, Savic S. A patient with systemic inflammation, relapsing polychondritis and pancytopaenia: VEXAS. Rheumatology (Oxford). 2022;61. 81. Ugwoke A, Keay J, Zarif A, Htut EEP. VEXAS syndrome in a patient with biopsy positive GCA and imaging proven large vessel vasculitis treated with tocilizumab. Ann Rheum Dis. 2023;82:1166. 82. Pozzi MR, De Matthaeis A, Bettini L, Campanella V, Riva L, Scali B, et al. VEXAS syndrome: two cases presenting with periorbital edema. Ann Rheum Dis. 2023;82:1167. 83. Galindo-Feria AS, Chatzidionysiou K. An unusual initial presentation of VEXAS syndrome. Ann Rheum Dis. 2023;82:1164-5. 84. Lacombe V, Beucher A, Urbanski G, Le Corre Y, Cottin L, Croué A, et al. Distinction between clonal and paraclonal cutaneous involvements in VEXAS syndrome. Exp Hematol Oncol. 2022;11:6. 85. Invited speaker presentation abstracts. Int J Rheum Dis. 2023;26:4–25. 86. Cordts I, Hecker JS, Gauck D, Park J, Härtl J, Günthner R, et al. Successful treatment with azacitidine in VEXAS syndrome with prominent myofasciitis. Rheumatology (Oxford). 2022;61:e117–9. 87. Medical dermatology. Australas J Dermatol. 2023;64:54–76. 88. Strasser B, Hoermann G, Haushofer A. UBA1 gene mutation establishes the diagnosis of VEXAS-syndrome in a patient with MDS. Clin Chem Lab Med. 2023;61(8):eA115–31. 89. Kunishita Y, Kirino Y, Tsuchida N, Maeda A, Sato Y, Takase-Minegishi K, et al. Case report: Tocilizumab treatment for VEXAS syndrome with relapsing polychondritis: A single-center, 1-year longitudinal observational study in japan. Front Immunol. 2022;13:901063. 90. Thomas B, Campagne J. Vexas: A new syndrome not always associated with hemopathy. EJHaem. 2022;3:235. 91. Fernandez JA, Patel BA, Gutierrez-Rodrigues F, Kusne Y, Lasho T, Finke C, et al. DNMT3A/TET2 mutant clonal hematopoiesis in vexas syndrome results in DNA hypomethylation and transcriptional activation of WT1 and MPL oncogenic pathways. Blood. 2022;140:11410–1. 92. Valor-Méndez L, Sticherling M, Zeschick M, Atreya R, Schmidt FD, Waldfahrer F, et al. VEXAS syndrome mimicking lupus-like disease. Rheumatology (Oxford). 2023;62:e271–2. 93. Stewart M, Williams S, Fifi-Mah A, Dehghan N. VEXAS syndrome: insights from a Canadian case series [abstract]. J Rheumatol. 2025;52(3):56. 94. Hines AS, Mohandesi NA, Lehman JS, Koster MJ, Cantwell HM, Alavi A, et al. Cutaneous involvement in VEXAS syndrome: clinical and histopathologic findings. Int J Dermatol. 2023;62:938–45. 95. Lucchino B, Delfino L, Patuzzo G, Finucci A, Ghellere F, Bortolotti ME, et al. SARS-CoV-2-induced venous thromboembolism in a patient with VEXAS syndrome. Rheumatol Adv Pract. 2023;7:rkad012. 96. McLean-Tooke A. VEXAS syndrome. Pathology. 2022;54:S24. 97. Koster M, Kourelis T, Reichard K, Kermani T, Beck D, Cardona DO, et al. Clinical heterogeneity of the VEXAS syndrome: a case series. Arthritis Rheumatol. 2021;73:1–4259. 98. Saha MK, Rivenbark J, Caceres-Nazario B, Buglioni A, Rubinstein SM. Interstitial inflammation and C3 glomerulopathy secondary to an autoinflammatory syndrome. J Am Soc Nephrol. 2023;34:291. 99. Beck DB, Wu Z, Patel BA, Ferrada MA, Sikora KA, Ombrello A, et al. Somatic mutations in a single residue of UBA1 cause vexas, a severe adult-onset rheumatic disease associated with myeloid dysplasia. Blood. 2020;136:36–7. 100. Beck D, Ferrada M, Sikora K, Ombrello A, Cardona DO, Balanda N, et al. Somatic mutations in a single residue of UBA1 cause VEXAS, a severe adult-onset rheumatic disease presenting as relapsing polychondritis, polyarteritis nodosa, or giant cell arteritis. Arthritis Rheumatol. 2020;72(Suppl 10):1-4231. 101. Zewar A, Zollinger-Read C, Dunkley L, Jefferis JM. Painful diplopia as an initial presentation of VEXAS syndrome. Neuroophthalmology. 2024;48:360–3. 102. Prabahran AA, Gutierrez-Rodrigues F, Ahmad S, Cowen EW, Darden I, Groarke EM, et al. Eosinophilia in vexas syndrome: Expanding hematologic phenotype. Blood. 2022;140:2607–8. 103. Moura MC, Baqir M, Tandon Y, Samec MJ, Reichard KK, Mangaonkar A, et al. Lung involvement in VEXAS syndrome. Arthritis Rheumatol. 2022;74(Suppl 0):3103–4. 104. Coattrenec Y, Brücker R, Matulis G, Müller B, Samii K, De Lorenzi C, et al. The VEXAS syndrome: report of 3 new cases. Swiss Med Wkly. 2021;151(Suppl):18S. 105. Leightell-Brown L, Sommer S. BG07 VEXAS syndrome without the V: a New Zealand case report. Br J Dermatol. 2023;188. 106. Barrios Millán G, Servera G, Sendagorta E, García Fernández E, Ruiz-Bravo E, Beato MJ. VEXAS: case report and characteristics of a new syndrome with neutrophilic dermatosis. Virchows Arch. 2022;481:S213. 107. Kusne Y, Fernandez J, Patnaik MM. Clonal hematopoiesis and VEXAS syndrome: survival of the fittest clones? Semin Hematol. 2021;58:226–9. 108. Ferrada M, Savic S, Alessi H, Ospina D, Wilson L, Goodspeed W, et al. Genotype and transfusion dependence predicts mortality in VEXAS syndrome, a newly described disease with overlap inflammatory and hematologic features. Arthritis Rheumatol. 2021;73:1–4259. 109. Borie R, Debray MP, Audemard A, Guedon A, Terriou L, Lacombe V, et al. Pleuropulmonary manifestations of VEXAS syndrome. Eur Respir J. 2022;60(Suppl 66):104. 110. Groarke E, Patel B, Dulau‐Florea A, Ferrada M, Lotter J, Darden I, et al. Patients with VEXAS syndrome due to somatic mutations in UBA1 present commonly with thrombosis [abstract]. Res Pract Thromb Haemost. 2021;5:e12591. 111. Kalantari K, Hernandez LPH, Bu L, Nasr SH, Cornell LD, Warrington K, et al. Plasma cell-rich acute interstitial nephritis in VEXAS: An under-recognized disease feature. J Am Soc Nephrol. 2023;34:459. 112. Plesa A, Gutrin J, Roumier C, Hayette S, Tigaud I, Huet S, et al. Correlation of clonal hematopoiesis in vexas syndrome with immunophenotype alterations in CD34+ HSPCs (hematopoietic stem progenitors cells): Multiparametric flow cytometry heterogeneity in CD34+CD38- stem cells fraction and upregulation of CD90 thy-1 by unsupervised strategy using PCA, tsnee and flowsom. Blood. 2022;140:4086–7. 113. Patel BA, Ferrada M, Quinn K, Freeman K, Darden I, Goodspeed W, et al. Clinical manifestations are evolving and progressive in patients with vexas syndrome. Blood. 2023;142:703. 114. Ahmad S, Ferrada M, Beck DB, Wilson LL, Grayson PC, Cowen EW. 156 Cutaneous spectrum of VEXAS syndrome. J Invest Dermatol. 2022;142:S27. 115. Chiaramida A, Obwar SG, Nordstrom AEH, Ericsson M, Saldanha A, Ivanova EV, et al. Sensitivity to targeted UBA1 inhibition in a myeloid cell line model of VEXAS syndrome. Blood Adv. 2023;7:7445–56. 116. Koster M, Herrera-Hernandez L, Bu L, Montes D, Nasr S, Cornell L, et al. Acute interstitial nephritis with plasma cell-rich infiltrate and acute kidney injury in VEXAS syndrome: an under-recognized disease feature. Arthritis Rheumatol. 2023;75:S524–7. 117. Deeb G. Conventional morphology still matters: the tales told by vacuoles from copper deficiency to VEXAS (case studies). Int J Lab Hematol. 2023;45:3–137. 118. Kirchen H, Thamm D, Deligiannis I, Weiner S. VEXAS syndrome: a single centre case series analysis of treating a rare novel entity. Oncol Res Treat. 2023;46:1–354. 119. Martín-Nares E, Vargas-Serafín C, Delgado-de la Mora J, Montante-Montes de Oca D, Grayson PC, Larios E, et al. Orbital and periorbital inflammation in VEXAS syndrome. Scand J Rheumatol. 2022;51:338–41. 120. Lynch F, Murray K, Leonard N, Sadlier M. A truly VEXing diagnosis. Br J Dermatol. 2023;188(Suppl 4):ljad113.028. 121. Ferrada M, Grayson P, Wilson L, Beck D, Goodspeed W, Darden I, et al. Evaluation for Allogeneic Hematopoietic Stem Cell Transplant Indications and Eligibility in a Prospective Cohort of Patients with VEXAS. Arthritis Rheumatol. 2022;74(Suppl 9):3101-2. 122. Beecher MB, Tong JY, Halliday LA, Hissaria P, Selva D. Recurrent orbital inflammation associated with VEXAS syndrome. Orbit. 2024;43:350–3. 123. Ford M, Khan T. Pos1580 a case series of vexas syndrome identified in the west midlands, United Kingdom. Ann Rheum Dis. 2023;82:1166–7. 124. Beecher M, Tong J, Halliday L, Hissaria P, Selva D. Recurrent dacryoadenitis associated with VEXAS syndrome. Clin Exp Ophthalmol. 2022;50(8):899-975. 125. Vergneault H, Picard C, Georgin-Lavialle S. Break down the barriers of auto-inflammation: How to deal with a monogenic auto-inflammatory disease and immuno-haematological features in 2022? Immunology. 2023;168:1–17. 126. Lucas J, Calev H, Cheeley J. VEXAS syndrome-a newly discovered autoimmune and hematologic overlap condition. J Hosp Med. 2023;18:S636. 127. Khoo T, Chin A, Walker J. The skin as a snapshot of systemic inflammation. Rheumatol Adv Pract. 2023;7:ii8-ii9. 128. Bindoli S, Bertoldo E, Doria A, Baggio C, Sfriso P. JAK inhibitors and VEXAS syndrome: a successfully case treated with filgotinib. Ann Rheum Dis. 2023;82:1166. 129. Topilow JS, Ospina Cardona D, Beck DB, Ferrada MA, McMahan ZH, Paik JJ. Novel genetic mutation in myositis-variant of VEXAS syndrome. Rheumatology (Oxford). 2022;61:e371–3. 130. Dave D, Malvar GL, Moeckel GW, Shirali AC. A vexing case. J Am Soc Nephrol. 2023;34:249–50. 131. Khider L, Templé M, Bally C, Spaeth A, Darnige L, Sanchez O, et al. Systematic search for the UBA1 mutation in men after a first venous thrombotic episode. Arch Cardiovasc Dis Suppl. 2023;15:182–3. 132. Collins JC, Balanda N, Magaziner SJ, English M, Ospina Cardona D, Patnaik MM, et al. novel disease-causing mutations in UBA1 reveal disease mechanisms in bone marrow failure and inflammation. Blood. 2022;140:2914–5. 133. Skowron F, Klepfisch L, Guillaume L, Godeneche J, Facile A, Gombert M, et al. VEXAS syndrome following COVID-19 mRNA vaccination. J Eur Acad Dermatol Venereol. 2023;37. 134. Nune A, Iyengar KP, Barman B, Manzo C. The VEXAS syndrome from rheumatology perspective: genomic DNA sequencing as available blueprint for diagnosing rheumatic diseases with overlapping haematological or dermatological findings. Clin Rheumatol. 2022;41:2917–8. 135. Kanagal-Shamanna R, Beck DB, Calvo KR. Clonal hematopoiesis, inflammation, and hematologic malignancy. Annu Rev Pathol. 2024;19:479–506. 136. Jachiet V, Ricard L, Hirsch P, Malard F, Zhao LP, Adès L, et al. Reduced peripheral blood myeloid cells in patients with VEXAS syndrome. Ann Rheum Dis. 2022;81:1157. 137. Beaumesnil S, Boucher S, Lavigne C, Urbanski G, Lacombe V. Ear, nose, throat, and bronchial involvements in VEXAS syndrome: Specifying the spectrum of clinical features. JAMA Otolaryngol Head Neck Surg. 2022;148:284–6. 138. Bruno A, Gurnari C, Greco R, Alexander T, Snowden J. Toward a clinical phenotype inspired diagnosis of autoimmune manifestations in VEXAS syndrome: a guide to UBA1-testing for the generalist. Bone Marrow Transplant. 2023;58:344-345. 139. Beck DB, Bodian DL, Shah V, Mirshahi UL, Kim J, Ding Y, et al. Genomic ascertainment for UBA1 variants and VEXAS syndrome: a population-based study. medRxiv. 2022:2022.07.27.22277962. 140. Barouqa MI, Ravindran A, Chen D, Oliveira JL, Olteanu H, Howard MT, et al. Histopathologic characterization of vexas syndrome. Blood. 2021;138:4656. 141. Tiwari V, Miller AT. Fever, rash, and shortness of breath in a 69-year-old. JAMA. 2024;331:698–9. 142. Uchino K, Kanasugi J, Enomoto M, Kitamura F, Tsuchida N, Uchiyama Y, et al. VEXAS syndrome. Int J Hematol. 2022;116:463–4. 143. Zhao L-P, Schell B, Sébert M, Kim R, Lemaire P, Boy M, et al. Prevalence of UBA1 mutations in MDS/CMML patients with systemic inflammatory and auto-immune disease. Leukemia. 2021;35:2731–3. 144. Tsuchida N, Kunishita Y, Uchiyama Y, Kirino Y, Takase-Minegishi K, Yoshimi R, et al. Pathogenic UBA1 Variants in Japanese Patients with Relapsing Polychondritis. Arthritis Rheumatol. 2021;73:1-4259. 145. Nyhuus Bendix Rasch M, Szabados F, Jensen JMB, Nielsen KO, Hauge EM, Troldborg A. Pos1348 patients with vexas diagnosed in a danish tertiary rheumatology setting have highly elevated inflammatory markers, macrocytic anemia, and negative autoimmune biomarkers. Ann Rheum Dis. 2022;81:1012–3. 146. Hamann I, Gunduz O. VEXAS Syndrome. Australas J Dermatol. 2023;64:54-76. 147. Fernández-Parrado M, Perandones-González H. RF-VEXAS syndrome: A new autoinflammatory disease. Actas Dermosifiliogr. 2023;114:T531–2. 148. Zhao L-P, Schell B, Kim R, Sébert M, Lemaire P, Boy M, et al. Prevalence of VEXAS syndrome in MDS/CMML patients with systemic inflammatory and auto-immune disease. Clin Lymphoma Myeloma Leuk. 2021;21:S337–8. 149. Euvrard R, Fournier T, Georgescu D, Bourbon E, Sujobert P, Lega JC, et al. VEXAS syndrome-related AA amyloidosis: a case report. Rheumatology (Oxford). 2021;61:e15–6. 150. Grayson PC, Perugino CA, Dinculescu VV, Ferry JA. Case 2-2022: A 70-year-old man with a recurrent left pleural effusion. N Engl J Med. 2022;386:274–83. 151. Kunishita Y, Kirino Y, Tsuchida N, Maeda A, Hirahara L, Nakajima H. Tocilizumab for VEXAS syndrome with relapsing polychondritis: extension study. Pediatr Rheumatol. 2022;20(Suppl 1):2. 152. Mekinian A, Bourguiba R, Terrier B, Olivier K, Comont T, Fenaux P, et al. Biologics and JAK inhibitors efficacy in VEXAS syndrome from French multicenter case series of 256 patients. Ann Rheum Dis. 2023;82:1955-6. 153. Kirino Y. What do we need to know about VEXAS syndrome. Int J Rheum Dis. 2023;26:4-25. 154. Sadawarte S, Nelson N, Vanhaeften R, Marthick J, Sharma A, Bapat A, et al. VEXAS syndrome identified on somatic NGS panel in a fraction of patients of myelodysplastic syndrome and myelodysplasia/myeloproliferative neoplasms with autoimmune and inflammatory disorders. Leuk Res. 2023;128:107154. 155. Gil-Lianes J, Luque-Luna M, Alamon-Reig F, Bosch-Amate X, Serra-Garcia L, Mascaró JM Jr. Sweet syndrome: Clinical presentation, malignancy association, autoinflammatory disorders and treatment response in a cohort of 93 patients with long-term follow-up. Acta Derm Venereol. 2023;103:adv18284. 156. Vetsiou E, Smith S, Forde K, McDonald S, Brian D, Khoo A. Severe adult-onset inflammatory disorder with associated UBA1 somatic mutation. Br J Dermatol. 2023;188(Suppl 4):ljad113.029. 157. Ferrada M, Sikora K, Lou Y, Wells K, Patel B, Ospina Cardona D, et al. Classification of patients with relapsing polychondritis based on somatic mutations in uba1. Ann Rheum Dis. 2021;80:49. 158. Gurnari C, Pascale MR, Vitale A, Diral E, Galossi E, Falconi G, et al. A cartography of UBA1 gene testing, epidemiology and clinical-genomic characteristics: the VEXAS/MDS Italian experience. Leuk Res. 2023;128:107148. 159. Robert M, Berleur M, Gaudemer A, Crow YJ, Frémond M-L, Sacré K. VEXAS syndrome: Expanding the clinical and molecular spectrum. Joint Bone Spine. 2023;90:105531. 160. Ashari KA, Hausmann JS, Dedeoglu F. Update on autoinflammatory diseases. Curr Opin Rheumatol. 2023;35:285–92. 161. Arnaud L, Costedoat-Chalumeau N, Mathian A, Sailler L, Belot A, Dion J, et al. French practical guidelines for the diagnosis and management of relapsing polychondritis. Rev Med Interne. 2023;44:282–94. 162. Mertz P, Sparks J, Kobrin D, Ogbonnaya SA, Sevim E, Michet C, et al. Relapsing polychondritis: Best Practice & Clinical Rheumatology. Best Pract Res Clin Rheumatol. 2023;37:101867. 163. Zakine E, Schell B, Battistella M, Vignon-Pennamen M-D, Chasset F, Mahévas T, et al. UBA1 variations in neutrophilic dermatosis skin lesions of patients with VEXAS syndrome. JAMA Dermatol. 2021;157:1349–54. 164. Bourbon E, Heiblig M, Gerfaud Valentin M, Barba T, Durel C-A, Lega JC, et al. Therapeutic options in VEXAS syndrome: insights from a retrospective series. Blood. 2021;137:3682–4. 165. Poulter JA, Collins JC, Cargo C, De Tute RM, Evans P, Ospina Cardona D, et al. Novel somatic mutations in UBA1 as a cause of VEXAS syndrome. Blood. 2021;137:3676–81. 166. Henrie R, Cherniawsky H, Marcon K, Zhao EJ, Marinkovic A, Pourshahnazari P, et al. Inflammatory diseases in hematology: a review. Am J Physiol Cell Physiol. 2022;323:C1121–36. 167. Heiblig M, Sujobert P. From vacuoles to VEXAS. Rheumatology (Oxford). 2023;62:3780–1. 168. Chen K-R. Cutaneous vasculitis in autoinflammatory diseases. J Dermatol. 2024;51:150–9. 169. Sikora KA, Wells KV, Bolek EC, Jones AI, Grayson PC. Somatic mutations in rheumatological diseases: VEXAS syndrome and beyond. Rheumatology (Oxford). 2022;61:3149–60. 170. Mekinian A, Zhao LP, Chevret S, Desseaux K, Pascal L, Comont T, et al. A Phase II prospective trial of azacitidine in steroid-dependent or refractory systemic autoimmune/inflammatory disorders and VEXAS syndrome associated with MDS and CMML. Leukemia. 2022;36:2739–42. 171. Poulter JA, Savic S. Genetics of somatic auto-inflammatory disorders. Semin Hematol. 2021;58:212–7. 172. Malcovati L. VEXAS: walking on the edge of malignancy. Blood. 2023;142:214–5. 173. Collins JC, Magaziner SJ, English M, Hassan B, Chen X, Balanda N, et al. Shared and distinct mechanisms of UBA1 inactivation across different diseases. EMBO J. 2024;43:1919–46. 174. Onuora S. Somatic mutations cause VEXAS syndrome. Nat Rev Rheumatol. 2021;17:1. 175. Alcedo PE, Gutierrez-Rodrigues F, Patel BA. Somatic mutations in VEXAS Syndrome and Erdheim-Chester disease: Inflammatory myeloid diseases. Semin Hematol. 2022;59:156–66. 176. Hagiya A, Siddiqi IN, Wang E, Lu CM. How I diagnose and manage VEXAS syndrome. Am J Clin Pathol. 2024;162:28–40. 177. Al-Hakim A, Mistry A, Savic S. Improving Diagnosis and Clinical Management of Acquired Systemic Autoinflammatory Diseases. J Inflamm Res. 2022;15:5739-55. 178. Patel N, Dulau-Florea A, Calvo KR. Characteristic bone marrow findings in patients with UBA1 somatic mutations and VEXAS syndrome. Semin Hematol. 2021;58:204–11. 179. Bindoli S, Baggio C, Doria A, Bertoldo E, Sfriso P. JAK inhibitors for the treatment of VEXAS syndrome. Exp Biol Med (Maywood). 2023;248:394–8. 180. Oo TM, Koay JTJ, Lee SF, Lee SMS, Lim XR, Fan BE. Thrombosis in VEXAS syndrome. J Thromb Thrombolysis. 2022;53:965–70. 181. Corty RW, Brogan J, Byram K, Springer J, Grayson PC, Bick AG. VEXAS-defining UBA1 somatic variants in 245,368 diverse individuals in the NIH All of us cohort. Arthritis Rheumatol. 2024;76:942–8. 182. Kucharz EJ. VEXAS syndrome: a newly discovered systemic rheumatic disorder. Reumatologia. 2023;61:123–9. 183. Heiblig M, Patel B, Jamilloux Y. VEXAS syndrome, a new kid on the block of auto-inflammatory diseases: A hematologist’s point of view. Best Pract Res Clin Rheumatol. 2023;37:101861. 184. Guerineau H, Kohn M, Al Hamoud A, Sellier J, Osman J, Cabannes-Hamy A. Could it be VEXAS? Ann Hematol. 2024;103:2169–71. 185. Corcia P. VEXAS syndrome extends the neurological complications of haemopathies. J Neurol Neurosurg Psychiatry. 2022;93:685. 186. Hernández-Rodríguez J, Mensa-Vilaró A, Aróstegui JI. Cambio de paradigma en las enfermedades autoinflamatorias monogénicas y las vasculitis sistémicas: el síndrome VEXAS. Med Clin (Barc). 2022;159:489–96. 187. Hernández-Rodríguez J, Nieścieruk J, Maślińska M. VEXAS syndrome: an adult-onset monogenic autoinflammatory disease with many unanswered questions. Reumatologia. 2023;61:83–5. 188. Himmelmann A, Brücker R. The VEXAS syndrome: Uncontrolled inflammation and macrocytic anaemia in a 77-year-old male patient. Eur J Case Rep Intern Med. 2021;8:002484. 189. Sakuma M, Blombery P, Meggendorfer M, Haferlach C, Lindauer M, Martens UM, et al. Novel causative variants of VEXAS in UBA1 detected through whole genome transcriptome sequencing in a large cohort of hematological malignancies. Leukemia. 2023;37:1080–91. 190. Onuora S. VEXAS syndrome more common than originally thought? Nat Rev Rheumatol. 2023;19:193. 191. Martínez-Diaz L, Morales-Angulo C. VEXAS syndrome and otolaryngology. Am J Otolaryngol. 2024;45:104216. 192. Raaijmakers MHGP, Hermans M, Aalbers A, Rijken M, Dalm VASH, van Daele P, et al. Azacytidine treatment for VEXAS syndrome. HemaSphere. 2021;5:e661. 193. Duminuco A, Vetro C, Markovic U, Di Raimondo F, Palumbo GAM. VEXAS-like syndrome: a potential new entity? Ann Hematol. 2022;101:1125–8. 194. Sockel K, Götze K, Ganster C, Bill M, Georgi J-A, Balaian E, et al. VEXAS syndrome: complete molecular remission after hypomethylating therapy. Ann Hematol. 2024;103:993–7. 195. Kobak S. What the genes tell us in the VEXAS syndrome? A rheumatologist’s perspective. Int J Rheum Dis. 2023;26:827–30. 196. Sirenko M, Bernard E, Creignou M, Domenico D, Farina A, Arango Ossa JE, et al. Molecular and clinical presentation of UBA1-mutated myelodysplastic syndromes. Blood. 2024;144:1221–9. 197. Heiblig M, Patel BA, Groarke EM, Bourbon E, Sujobert P. Toward a pathophysiology inspired treatment of VEXAS syndrome. Semin Hematol. 2021;58:239–46. 198. Matsumoto H, Fujita Y, Fukatsu M, Ikezoe T, Yokose K, Asano T, et al. Case report: Coexistence of multiple myeloma and auricular chondritis in VEXAS syndrome. Front Immunol. 2022;13:897722. 199. Barba T, Jamilloux Y, Durel C-A, Bourbon E, Mestrallet F, Sujobert P, et al. VEXAS syndrome in a woman. Rheumatology (Oxford). 2021;60:e402–3. 200. Midtvedt Ø, Stray-Pedersen A, Andersson H, Gunnarsson R, Tveten K, Ali MM, et al. A man in his sixties with chondritis and bone marrow failure. Tidsskr Nor Laegeforen. 2022;142(4). 201. Collantes-Rodríguez C, Jiménez-Gallo D, de la Varga-Martínez R, Mora-López F, Garrastazul-Sánchez MP, Linares-Barrios M. Vexas syndrome successfully treated with canakinumab. J Dtsch Dermatol Ges. 2023;21:69–70. 202. Templé M, Duroyon E, Croizier C, Rossignol J, Huet T, Friedrich C, et al. Atypical splice-site mutations causing VEXAS syndrome. Rheumatology (Oxford). 2021;60:e435–7. 203. Grayson PC, Beck DB, Ferrada MA, Nigrovic PA, Kastner DL. VEXAS syndrome and disease taxonomy in rheumatology. Arthritis Rheumatol. 2022;74:1733–6. 204. Haines P, Pullarkat S, Said J. VEXAS syndrome: Vacuoles in myeloid, erythroid, and lymphoid lineages. Int J Lab Hematol. 2024;46:8–9. 205. Cardoneanu A, Rezus II, Burlui AM, Richter P, Bratoiu I, Mihai IR, et al. Autoimmunity and autoinflammation: Relapsing polychondritis and VEXAS syndrome challenge. Int J Mol Sci. 2024;25:2261. 206. Pozdniakova H, Vedire A, Kadakia A, Imburgio S, Bajwa R, Gupta V, et al. A double hit to ubiquitination leading to a new diagnosis of VEXAS syndrome. J Med Cases. 2023;14:327–31. 207. Raman A, Damodaran A. Clinical images: Ureteritis in VEXAS syndrome. ACR Open Rheumatol. 2024;6:333. 208. Fernández-Parrado M, Perandones-González H. Foro para residentesFR- Síndrome Vexas: Una nueva enfermedad autoinflamatoria. Actas Dermosifiliogr. 2023;114:531–2. 209. Vu KT, Wolfe RM, Lambird JE, Maracaja DLV. A case of VEXAS syndrome presenting with unusual bone marrow granulomas: a diagnostic dilemma. BMC Rheumatol. 2023;7:18. 210. Grosse A, Salehi T, Callary M, Hecker JR, Hissaria P. VEXAS syndrome causing fever of unknown origin. Med J Aust. 2022;217:129–30. 211. Ciferska H, Gregová M, Klein M, Šenolt L, Soukupová Maaloufová J, Pavelka K, et al. VEXAS syndrome: a report of three cases. Clin Exp Rheumatol. 2022;40:1449. 212. Al-Hakim A, Cull A, Topping J, Nadat F, Milek J, Alhefzi R, et al. Recovery of bone marrow function in VEXAS syndrome-potential role for romiplostim. HemaSphere. 2023;7:e934. 213. Karadeniz H, Cerit M, Avanoğlu Güler A, Tufan A, Kanthi Y. Venous inflammation might be one of the features of VEXAS syndrome and associated thrombosis. Rheumatology (Oxford). 2023;62:e269–70. 214. Lacombe V, Prevost M, Bouvier A, Thépot S, Chabrun F, Kosmider O, et al. Vacuoles in neutrophil precursors in VEXAS syndrome: diagnostic performances and threshold. Br J Haematol. 2021;195:286–9. 215. Fenu EM, Bagg A. VEXAS’d by lymphadenopathy: an unusual case of extramedullary hematopoiesis. Blood. 2023;141:2784. 216. Louvrier C, Awad F, Amselem S, Lipsker D, Giurgea I. Absence of NLRP3 somatic mutations and VEXAS-related UBA1 mutations in a large cohort of patients with Schnitzler syndrome. Allergy. 2022;77:3435–6. 217. Stubbins RJ, McGinnis E, Johal B, Chen LY, Wilson L, Cardona DO, et al. VEXAS syndrome in a female patient with constitutional 45,X (Turner syndrome). Haematologica. 2022;107:1011–3. 218. Echerbault R, Bourguiba R, Georgin-Lavialle S, Lavigne C, Ravaiau C, Lacombe V. Comparing clinical features between males and females with VEXAS syndrome: data from literature analysis of patient reports. Rheumatology (Oxford). 2024;63:2694–700. 219. Fanlo P, Román ML de S, Fonollosa A, Ilarramendi J, Heras H, Grayson P. Episcleritis and periorbital edema secondary to VEXAS syndrome. Arch Soc Esp Oftalmol (Engl Ed). 2023;98:607–10. 220. Zakine È, Papageorgiou L, Bourguiba R, Mekinian A, Terrier B, Kosmider O, et al. Clinical and pathological features of cutaneous manifestations in VEXAS syndrome: A multicenter retrospective study of 59 cases. J Am Acad Dermatol. 2023;88:917–20. 221. Holmes A, Thant A, Correy R, Vilain R. Inflammatory pseudotumour arising secondary to VEXAS syndrome. Pathology. 2023;55:161–3. 222. Compton LA, Jones HA, Vinyard CA, Lee Y-S, Walter MJ, Yokoyama CC. Progressive, edematous plaques, mild pancytopenia, and inflammation. JAAD Case Rep. 2023;42:109–12. 223. Manzoni M, Bosi A, Fabris S, Lionetti M, Salerio S, Migliorini AC, et al. Clinical, morphological and clonal progression of VEXAS syndrome in the context of myelodysplasia treated with azacytidine. Clin Hematol Int. 2022;4:52–5. 224. Gurnari C, Koster L, Baaij L, Heiblig M, Yakoub-Agha I, Collin M, et al. Allogeneic hematopoietic cell transplantation for VEXAS syndrome: results of a multicenter study of the EBMT. Blood Adv. 2024;8:1444–8. 225. Nicholson LT, Madigan LM. Working towards a better understanding of VEXAS syndrome. Br J Dermatol. 2022;186:392–3. 226. Oganesyan A, Jachiet V, Chasset F, Hirsch P, Hage-Sleiman M, Fabiani B, et al. VEXAS syndrome: still expanding the clinical phenotype. Rheumatology (Oxford). 2021;60:e321–3. 227. Fan BE, Cao L, Gallardo CA, Lee SMS, Koh LW, Goh LL, et al. Myeloid and lymphoid vacuolation in VEXAS syndrome. Am J Hematol. 2021;96:1056–7. 228. Faurel A, Heiblig M, Kosmider O, Cornillon J, Boudou L, Guyotat D, et al. Recurrent mutations of the active adenylation domain of UBA1 in atypical form of VEXAS syndrome. HemaSphere. 2023;7:e868. 229. Tsourveloudis I, Georgiadi EC, Vatalis G, Kotsi P. Case report of a patient with VEXAS syndrome. Medicine (Baltimore). 2023;102:e36738. 230. Sánchez-Hernández BE, Calderón-Espinoza I, Martín-Nares E. Challenging the paradigm: a case of early-onset VEXAS syndrome. Rheumatology (Oxford). 2024;63:e99–100. 231. Oka H, Sumitomo S, Nishikubo M, Yamashita D, Maruoka H, Shiroishi Y, et al. VEXAS syndrome with granulomatosis with polyangiitis manifestation: retained in remission using methotrexate and infliximab. Rheumatology (Oxford). 2024;63:e110–2. 232. Itagane M, Teruya H, Kato T, Tsuchida N, Maeda A, Kirino Y, et al. Clinical images: VEXAS syndrome presenting as treatment-refractory polyarteritis nodosa. Arthritis Rheumatol. 2022;74:1863–4. 233. Ospina Cardona D, Rodriguez-Pinto I, Iosim S, Bonet N, Mensa-Vilaro A, Wong M-K, et al. Description of a novel splice site variant in UBA1 gene causing VEXAS syndrome. Rheumatology (Oxford). 2024;63:2897–902. 234. Olteanu H, Patnaik M, Koster MJ, Herrick JL, Chen D, He R, et al. Comprehensive morphologic characterization of bone marrow biopsy findings in a large cohort of patients with VEXAS syndrome: A single-institution longitudinal study of 111 bone marrow samples from 52 patients. Am J Clin Pathol. 2024;161:609–24. 235. Bert-Marcaz C, Briantais A, Faucher B, Corazza G, Ebbo M, Attarian S, et al. Expanding the spectrum of VEXAS syndrome: association with acute-onset CIDP. J Neurol Neurosurg Psychiatry. 2022;93:797–8. 236. Mangaonkar AA, Langer KJ, Lasho TL, Finke C, Litzow MR, Hogan WJ, et al. Reduced intensity conditioning allogeneic hematopoietic stem cell transplantation in VEXAS syndrome: Data from a prospective series of patients. Am J Hematol. 2023;98:E28–31. 237. Thomas VT, Penmetcha M. Myelodysplastic syndrome associated with auto-immune inflammatory disease in VEXAS syndrome. J Hematol (Brossard). 2021;10:274–6. 238. Johansen MM, El Fassi D, Nielsen CTH, Krintel SB, Graudal N, Hansen JW. Treatment experiences with focus on IL-6R inhibition in patients with VEXAS syndrome and a case of remission with azacytidine treatment. Rheumatology (Oxford). 2025;64:826–30. 239. Legeas C, Saucereau J, Saraux A, Schoenlaub P. VEXAS syndrome: A first case without any haematological abnormalities. Joint Bone Spine. 2023;90:105473. 240. Kirino Y, Takase-Minegishi K, Tsuchida N, Hirahara L, Kunishita Y, Yoshimi R, et al. Tocilizumab in VEXAS relapsing polychondritis: a single-center pilot study in Japan. Ann Rheum Dis. 2021;80:1501–2. 241. Boret M, Malfait T. Case report: diagnosis of VEXAS syndrome in a patient with therapy-resistant large vessel vasculitis. Acta Clin Belg. 2024;79:143–7. 242. Lytle A, Bagg A. VEXAS: a vivid new syndrome associated with vacuoles in various hematopoietic cells. Blood. 2021;137:3690. 243. Horton RK, Zheng G. A case of VEXAS syndrome with subtle morphologic findings. Blood. 2021;138:1378. 244. Lacombe V, Kosmider O, Prévost M, Lavigne C, Urbanski G. Severe joint involvement in VEXAS syndrome: A case report. Ann Intern Med. 2021;174:1025–7. 245. Strasser B, Haushofer A. The first case of VEXAS syndrome in Austria. Clin Chem Lab Med. 2023;61:e187–8. 246. Archambeaud A, Le Dreau C, Bigot A, Kosmider O, Taleb A, Boucher L, et al. Trismus as a new feature of VEXAS syndrome. Rheumatology (Oxford). 2024;63:e258–60. 247. Rabut A, Jasserand L, Richard C, Dumas C, Mestrallet F, Bourbon E, et al. Quantitative assessment of vacuolization of myeloid precursors in VEXAS syndrome. HemaSphere. 2023;7:e828. 248. Djerbi N, Zimmermann K, Roncador M, Becker MO, Manz MG, Balabanov S. Intrapatient competition of VEXAS syndrome and CML clones. Blood Adv. 2023;7:6815–8. 249. Rieu J-B, El Kassir A, Largeaud L, Dion J, Comont T, Mansat-De Mas V. Characteristic vacuolisation of granulocytic and erythroid precursors associated with VEXAS syndrome. Br J Haematol. 2021;194:8. 250. Ribereau-Gayon E, Heiblig M, Bourbon E, Sujobert P, Harou O, Theillac C, et al. Atypical extensive lupus tumidus-like eruption as an early presentation of VEXAS syndrome. Int J Dermatol. 2022;61:e89–91. 251. Escoda T, Farnault L, Gallard J, Marceau-Renaut A, Attarian S, Delmont E. Azacitidine, a therapeutic option in Lewis and Sumner syndrome associated with VEXAS syndrome. Rev Neurol (Paris). 2022;178:1109–11. 252. Lucchino B, Finucci A, Ghellere F, Bortolotti ME, Tedesco A, Lombardi S. Influence of HLA polymorphisms on clinical features of VEXAS syndrome: a potential epistatic mechanism. Rheumatology (Oxford). 2022;62:e7–8. 253. Hage-Sleiman M, Lalevée S, Guermouche H, Favale F, Chaquin M, Battistella M, et al. Dominance of an UBA1 mutant clone over a CALR mutant clone: from essential thrombocytemia to VEXAS. Haematologica. 2021;106:3245–8. 254. Li P, Venkatachalam S, Ospina Cordona D, Wilson L, Kovacsovics T, Moser KA, et al. A clinical, histopathological, and molecular study of two cases of VEXAS syndrome without a definitive myeloid neoplasm. Blood Adv. 2022;6:405–9. 255. Pàmies A, Ferràs P, Bellaubí-Pallarés N, Giménez T, Raventós A, Colobran R. VEXAS syndrome: relapsing polychondritis and myelodysplastic syndrome with associated immunoglobulin A vasculitis. Rheumatology (Oxford). 2022;61:e69–71. 256. Yoon JG, Lee S, Kim S, Kim MJ, Chang YH, Park JK, et al. The first Korean case of VEXAS syndrome caused by a UBA1 somatic variant. Ann Lab Med. 2023;43:217–20. 257. D’Angelo G. Hematopoietic cells vacuolation, not always a reactive event. The VEXAS syndrome. Int J Lab Hematol. 2023;45:e15–6. 258. Al-Hakim A, Kulasekararaj A, Norouzi M, Medlock R, Patrick F, Cargo C, et al. S56F UBA1 variant is associated with haematological predominant subtype of VEXAS. Br J Haematol. 2023;203:331–5. 259. Diral E, Campochiaro C, Tomelleri A, Bergonzi GM, Pizzano U, Ponzoni M, et al. Case report: Cytopenias in VEXAS syndrome - a WHO 2022 based approach in a single-center cohort. Front Immunol. 2024;15:1354130. 260. Delplanque M, Mekinian A, Georgin-Lavialle S. Commentary: “case report: A rare case of elderly-onset adult onset still”s disease in a patient with systemic lupus erythematous’. Front Immunol. 2022;13:876477. 261. Gurnari C, Mannion P, Pandit I, Pagliuca S, Voso MT, Maciejewski JP, et al. UBA1 screening in sweet syndrome with hematological neoplasms reveals a novel association between VEXAS and chronic myelomonocytic leukemia. HemaSphere. 2022;6:e775. 262. Koster MJ, Ghaffar U, Kermani TA, Patnaik MM, Go RS, Mangaonkar AA, et al. Antineutrophil cytoplasmic antibody-associated vasculitis and VEXAS syndrome: comment on the article by Muratore et al. Arthritis Rheumatol. 2023;75:1490–2. 263. Fan BE, Sum CLL, Leung BPL, Ang MK, Lim XR, Lee SSM, et al. VEXAS syndrome and thrombosis: Findings of inflammation, hypercoagulability, and endothelial dysfunction. Semin Thromb Hemost. 2024;50:897–901. 264. Lee SMS, Fan BE, Lim JH-L, Goh LL, Lee JSS, Koh LW. A case of VEXAS syndrome manifesting as Kikuchi-Fujimoto disease, relapsing polychondritis, venous thromboembolism and macrocytic anaemia. Rheumatology (Oxford). 2021;60:e304–6. 265. Sallés M, Fustà X, Creus L, Esquius M, Mínguez S. Systemic lupus erythematosus in the elderly finally diagnosed as VEXAS syndrome. Clin Exp Rheumatol. 2024;42:764–5. 266. Lohaus N, Schaab J, Schaer D, Balabanov S, Huellner MW. VEXAS syndrome with tracheal involvement but absence of vasculitis in FDG PET/CT. Clin Nucl Med. 2023;48:e444–5. 267. Fahmy LM, Schreidah CM, Lapolla BA, Magro CM, Geskin LJ. VEXAS syndrome presenting as refractory cutaneous Kikuchi disease-like inflammatory pattern responding to tofacitinib. JAAD Case Rep. 2023;38:136–40. 268. Yılmaz U, Güner S, Eşkazan T, Demiröz AS, Kurtuluş G, Bahar F, et al. Kikuchi Fujimoto disease as the presenting component of VEXAS syndrome: a case report of a probable association. Clin Rheumatol. 2022;41:3589–92. 269. Allison DR, Dholaria B, Kishtagari A, Mohan S, Steigelfest E, Shaver AC, et al. Distinct bone marrow findings associated with a noncanonical UBA1 variant in VEXAS syndrome. Am J Hematol. 2024;99:1400–2. 270. Magnol M, Couvaras L, Degboé Y, Delabesse E, Bulai-Livideanu C, Ruyssen-Witrand A, et al. VEXAS syndrome in a patient with previous spondyloarthritis with a favourable response to intravenous immunoglobulin and anti-IL17 therapy. Rheumatology (Oxford). 2021;60:e314–5. 271. Sakuma M, Tanimura A, Yasui S, Ishiguro K, Kobayashi T, Ohshiro Y, et al. A Case of polychondritis-onset refractory organizing pneumonia with cytopaenia diagnosed as VEXAS syndrome: the disease course of 7 years. Rheumatology (Oxford). 2021;60:e356–9. |
| Wrong language  (n = 3) | 1. Stubbins RJ, Cherniawsky H, Chen LYC, Nevill TJ, et al. Innovations en génomique pour les maladies non diagnostiquées: le syndrome VEXAS (vacuoles intracytoplasmiques dans les progéniteurs médullaires, E1 ubiquitine ligase, liée à l'X, syndrome auto-inflammatoire, mutation somatique). CMAJ. 2022;194(34):E1179-83. 2. Ripke A, Münchau A, von Bubnoff N, Jendrek S, Kopelke S, Kümpers C, et al. B-Symptomatik bei unklarer mediastinaler Lymphadenopathie: Fallbericht eines 72-jährigen Patienten mit VEXAS-Syndrom. Inn Med (Heidelb). 2023;64(8):810-4. 3. Zeeck M, Kötter I, Krusche M. VEXAS-Syndrom. Z Rheumatol. 2022;81(9):782-6. |

**Supplementary Table 3.** Data extraction form for analytical observational studies of VEXAS syndrome in the critical care setting

| **No** | **Author and Year** | **Country** | **Aim** | **Population** | **Sample size** | **Key findings relevant to the scoping review questions** |
| --- | --- | --- | --- | --- | --- | --- |
| 1 |  |  |  |  |  |  |
| 2 |  |  |  |  |  |  |
| 3 |  |  |  |  |  |  |
| 4 |  |  |  |  |  |  |
| 5 |  |  |  |  |  |  |
| 6 |  |  |  |  |  |  |

**Supplementary Table 4.** Data extraction form for case reports and case series of VEXAS syndrome in the critical care setting

| **No** | **Author and year** | **Age** | **Gender** | **Key findings relevant to the scoping review questions** | **ICU admission** | **Outcome** |
| --- | --- | --- | --- | --- | --- | --- |
| 1 |  |  |  |  |  |  |
| 2 |  |  |  |  |  |  |
| 3 |  |  |  |  |  |  |
| 4 |  |  |  |  |  |  |
| 5 |  |  |  |  |  |  |
| 6 |  |  |  |  |  |  |

**Supplemental Table 5.** Individual cases of VEXAS syndrome managed in the critical care setting

**Abbreviations:** COVID-19, coronavirus disease 2019; ESBL, Extended-Spectrum Beta-Lactamase; HLH, hemophagocytic lymphohistiocytosis; VEXAS, Vacuoles, E1 enzyme, X-linked, Autoinflammatory, Somatic mutations

*Separate clinical episodes occurring at different times. **Information obtained through direct correspondence with the author.

| **Author and year** | **Age** | **Gender** | **Clinical characteristics and treatment** | **ICU admission** | **Outcome** |
| --- | --- | --- | --- | --- | --- |
| van der Made, et al. 2022 [1] | 76 | Male | Sepsis due to urinary tract infection caused by gram-negative rods  Treatment: antibiotic therapy | N/A | Survival |
|  | 79 | Male | Jejunum perforation  Treatment: infection source control management (operation) | N/A | Death |
|  | 81 | Male | Ileum perforation  Treatment: antibiotic therapy | N/A | Death |
|  | 71 | Male | Sepsis by *Streptococcus pneumoniae**; septic shock with lactic acidosis and respiratory failure* | Yes | Death |
|  | 71 | Male | Cardiovascular event (No details provided) | N/A | Death |
|  | 47 | Male | Acute respiratory distress syndrome | N/A | Survival |
|  | 70 | Male | Sepsis due to complicated urinary tract infection and respiratory failure | N/A | Death |
|  | 56 | Male | Severe influenza pneumonia | Yes | Survival |
| Guerrero-Bermúdez, et,al. 2022 [2] | 72 | Male | Prominent supraglottic larynx edema due to polychondritis  Treatment: corticosteroids | N/A | Survival |
| Belicard, et al. 2023 [3] | 70 | Male | Cardiac arrest due to hypoxia from airway obstruction caused by a swallowing disorder and retro-cricoarytenoid edema  Treatment: mechanical ventilation and targeted temperature management | Yes | Survival |
| Zisapel, et al. 2024 [4] | 82 | Male | Seizure and altered mental status due to cerebral sinus vein thrombosis  Treatment: mechanical ventilation, anticoagulation for thrombosis, high-dose intravenous corticosteroids, antiepileptics, endovascular thrombectomy and intracranial pressure lowering agents | N/A | Death |
| Rasch, et al. 2022 [5] | 74 | Male | Heart failure | N/A | Death |
| Muratore, et al. 2022 [6] | 74 | Male | Pulmonary complications (No details provided) | N/A | Death |
| Myint, et al. 2023 [7] | 76 | Male | Recurrent chest infections, congestive cardiac failure, and bilateral pulmonary embolism | Yes | Survival |
| Varadarajan, et al. 2023 [8] | 69 | Male | Subglottic edema | Yes | Survival |
| Estes, et al. 2023 [9] | 63 | Male | Anaphylactic shock due to azacitidine | N/A | Survival |
| Battipaglia, et al. 2023 [10] | 61 | Male | Sepsis caused by ESBL-producing *Klebsiella pneumoniae* and rectorrhagia*; sepsis caused by ESBL-producing *Klebsiella pneumoniae* with acute kidney injury, acute diverticulitis, and colitis-induced gastrointestinal bleeding*; sepsis by *Candida Cruzei* and *Staphylococcus Haemoliticus** | Yes | Survival |
| Diarra, et al. 2022 [11] | 55 | Male | Hypoxia due to severe pneumonia | N/A | Death |
| Yamaguchi, et al. 2023 [12] | 78 | Male | High-output heart failure due to left common iliac arteriovenous fistula  Treatment: operation for the iliac aneurysm (coil embolisation and endovascular aortic repair) | N/A | Survival |
| Staels, et al. 2021 [13] | 69 | Male | Hemophagocytic lymphohistiocytosis  Treatment: corticosteroids, siltuximab, rituximab and sirolimus | N/A | Survival |
| Matsumoto, et al. 2022 [14] | 60 | Male | Multiple organ failure associated with hyperinflammatory conditions  Treatment: high-dose corticosteroids, cyclosporine A, plasmapheresis and tocilizumab | N/A | Death |
| Więsik-Szewczyk, et,al. 2024 [15] | 69 | Male | Sepsis and multiple organ failure | N/A | Death |
| Freitas, et al. 2023 [16] | 76 | Female | Systemic inflammatory reaction with pulmonary involvement | N/A | Death |
| Johnsson, et al. 2024 [17] | 77 | Male | Altered mental status due to encephalitis  Treatment: mechanical ventilation, corticosteroids and azathioprine | N/A | Death |
| Raj, et al. 2023 [18] | 71 | Male | Sepsis (No details provided) | N/A | Death |
|  | 77 | Male | Sepsis due to cholecystitis | N/A | Death |
| Zaimoku, et al. 2023 [19] | 51 | Male | Interstitial pneumonia | N/A | Death |
| Al-Hakim, et al. 2022 [20] | 51 | Male | Sepsis and multiple organ failure | N/A | Death |
|  | 67 | Male | HLH, encephalitis, *Epstein–Barr virus* reactivation, and recurrent bacterial infections | N/A | Survival |
|  | 62 | Male | Sepsis (No details provided) | N/A | Death |
| Sardarli, et al. 2024 [21] | 47 | Male | Acute kidney injury (serum creatinine of 8.85 mg/dL) and volume overload  Treatment: hemodialysis | N/A | Survival |
| van Leeuwen‐Kerkhoff, et al. 2022 [22] | 47 | Male | Acute respiratory distress syndrome  Treatment: corticosteroids, anakinra | N/A | Survival |
| Ronsin, et al. 2022 [23] | 72 | Male | Acute kidney injury (serum creatinine of 15.2 mg/dL)  Treatment: corticosteroids, hemodialysis | N/A | Survival |
| Nguyen, et al. 2022 [24] | 63 | Male | Recurrent respiratory decompensation due to respiratory tract infection | N/A | Survival |
| Tosato, et al. 2023 [25] | N/A | Male | Experience of intensive care (No details provided) | Yes | Death |
| Austestad, et al. 2023 [26] | 69 | Male | Sepsis (No details provided) | N/A | Survival |
| Abumanhal, et al. 2024 [27] | 81 | Male | Seizure and consciousness impairment due to cerebral sinus vein thrombosis  Treatment: anticoagulation | N/A | Death |
| Riescher, et al. 2024 [28] | 65 | Male | Septic shock due to urinary tract infection by *Enterococcus faecium* | N/A | Death |
|  | 68 | Male | Cardiac failure | N/A | Death |
| Shimizu, et al. 2022 [29] | 71 | Male | Sepsis due to cellulitis*; Seizure due to brain abscesses by *Nocardia**; infective endocarditis by *Escherichia coli**; Sepsis due to sigmoid diverticulum perforation and necrotizing cholecystitis*  Treatment: antibiotic therapy and source control for infections | N/A | Death |
| Mayo-Juanatey, et al. 2024 [30] | 81 | Male | Sepsis due to urinary tract infection | N/A | Death |
| Islam, et al. 2022 [31] | 67 | Male | Suspected septic shock | Yes | Survival |
| Kao, et al. 2022 [32] | 56 | Male | HLH associated with *Epstein‐Barr virus*, respiratory failure with bilateral lung consolidations, and sepsis due to bacteremia by *Pseudomonas aeruginosa*  Treatment: corticosteroids, anakinra, rituximab and ruxolitinib for HLH | N/A | Death |
| Miyoshi, et al. 2023 [33] | 52 | Male | HLH associated with *Epstein‐Barr virus*, febrile neutropenia due to *Klebsiella pneumoniae* bacteremia, and right renal abscess with *Enterococcus faecium* and *Staphylococcus haemolyticus* bacteremia  Treatment: cyclophosphamide, hydroxydaunorubicin, oncovin, prednisone (CHOP regimen), etoposide, intravenous immunoglobulin and plasma exchange for HLH and antibiotic therapy | N/A | Survival |
| Pinto, et al. 2023 [34] | 78 | Male | Sepsis (No details provided) | N/A | Death |
| Wilson, et al. 2022 [35] | 66 | Male | Septic and cardiogenic shock due to pneumonia and stress cardiomyopathy with cardiac arrest  Treatment: fluid resuscitation and vasopressor for shock, antibiotic therapy and mechanical ventilation | Yes | Survival |
| Sofi, et al. 2024 [36] | late 30s | Male | Diffuse alveolar hemorrhage  Treatment: high-dose corticosteroids | N/A | Survival |
| Amakusa, et al. 2024 [37] | 67 | Male | Sepsis due to febrile neutropenia by gram-negative rods | N/A | Death |
| Delplanque, et al. 2021 [38] | 73 | Male | Sepsis and heart failure | N/A | Death |
| Ciprian, et al. 2022 [39] | 56 | Male | Distributive shock due to VEXAS syndrome**  Treatment: fluid resuscitation and vasopressor for shock, antibiotic therapy and corticosteroids | N/A | Survival |
| Grey, et al. 2021 [40] | 77 | Male | HLH associated with *Campylobacter jejuni* bacteremia due to soft tissue infection  Treatment: high-dose corticosteroids | N/A | Death |
| Suárez-Díaz, et.al. 2024 [41] | 70 | Male | Recurrent infection due to *Listeria monocytogenes* meningitis, urinary tract infection, and *Pneumocystis jiroveci* pneumonia | N/A | Death |
| Sharma, et al. 2022 [42] | 70 | Male | Respiratory failure due to COVID-19  Treatment: mechanical ventilation and corticosteroids | N/A | Survival |
| Salehi, et al. 2023 [43] | 69 | Male | Sepsis due to pneumonia, pulmonary hemorrhage, and colitis by *Cytomegalovirus*, *Clostridium difficile* and *Campylobacter jejuni*  Treatment: antibiotic therapy and corticosteroids | N/A | Death |
| Kusne, et al. 2024 [44] | 77 | Male | Cardiac arrest due to myocardial infarction  Treatment: coronary stent implantation and vasopressor | N/A | Death |

**References**

1. van der Made CI, Potjewijd J, Hoogstins A, Willems HPJ, Kwakernaak AJ, de Sevaux RGL, et al. Adult-onset autoinflammation caused by somatic mutations in UBA1: A Dutch case series of patients with VEXAS. J Allergy Clin Immunol. 2022;149:432–9.e4.

2. Guerrero-Bermúdez CA, Cardona-Cardona AF, Ariza-Parra EJ, Arostegui JI, Mensa-Vilaro A, Yague J, et al. Vacuoles, E1 enzyme, X-linked, autoinflammatory, somatic syndrome (VEXAS syndrome) with prominent supraglottic larynx involvement: a case-based review. Clin Rheumatol. 2022;41:3565–72.

3. Belicard F, Belhomme N, Bouzy S, Saillard C, Nedelec F, Mear J-B, et al. Vacuoles, E1 enzyme, X-linked, autoinflammatory, and somatic syndrome in the intensive care unit: a case report. J Med Case Rep. 2023;17:314.

4. Zisapel M, Seyman E, Molad J, Hallevi H, Mauda-Havakuk M, Jonas-Kimchi T, et al. Case report: Cerebral sinus vein thrombosis in VEXAS syndrome. Front Med (Lausanne). 2024;11:1377768.

5. Rasch MNB, Szabados F, Jensen JMB, Nielsen KO, Hauge E-M, Troldborg A. Patients with VEXAS diagnosed in a Danish tertiary rheumatology setting have highly elevated inflammatory markers, macrocytic anaemia and negative autoimmune biomarkers. RMD Open. 2022;8:e002492.

6. Muratore F, Marvisi C, Castrignanò P, Nicoli D, Farnetti E, Bonanno O, et al. VEXAS syndrome: A case series from a single-center cohort of Italian patients with vasculitis. Arthritis Rheumatol. 2022;74:665–70.

7. Myint K, Patrao N, Vonica O, Vahdani K. Recurrent superior orbital fissure syndrome associated with VEXAS syndrome. J Ophthalmic Inflamm Infect. 2023;13:39.

8. Varadarajan A, Verghese RM, Tirlangi PK, Dass J, Soneja M, Seth T. VEXAS syndrome (vacuoles, E1 enzyme, X-linked, autoinflammatory, somatic). QJM. 2023;116:313–5.

9. Estes J, Malus M, Wilson L, Grayson PC, Maz M. A case of VEXAS: Vacuoles, E1 enzyme, X-linked, autoinflammatory, somatic syndrome with co-existing DNA (cytosine-5)-methyltransferase 3A mutation complicated by localized skin reaction to tocilizumab and azacitidine. Cureus. 2023;15:e39906.

10. Battipaglia G, Vincenzi A, Falconi G, Fiore A, D’Agostino F, Iannotta R, et al. New scenarios in Vacuoles, E1 enzyme, X linked, Autoinflammatory, Somatic (VEXAS) syndrome: Evolution from myelodysplastic syndrome to acute myeloid leukemia. Curr Res Transl Med. 2023;71:103386.

11. Diarra A, Duployez N, Fournier E, Preudhomme C, Coiteux V, Magro L, et al. Successful allogeneic hematopoietic stem cell transplantation in patients with VEXAS syndrome: a 2-center experience. Blood Adv. 2022;6:998–1003.

12. Yamaguchi H, Kobayashi D, Nakamura G, Aida R, Horii Y, Okamoto T, et al. Acute heart failure due to left common iliac arteriovenous fistula: A case of VEXAS syndrome. Mod Rheumatol Case Rep. 2023;7:327–33.

13. Staels F, Betrains A, Woei-A-Jin FJSH, Boeckx N, Beckers M, Bervoets A, et al. Case report: VEXAS syndrome: From mild symptoms to life-threatening macrophage activation syndrome. Front Immunol. 2021;12:678927.

14. Matsumoto H, Asano T, Tsuchida N, Maeda A, Yoshida S, Yokose K, et al. Behçet’s disease with a somatic UBA1 variant:Expanding spectrum of autoinflammatory phenotypes of VEXAS syndrome. Clin Immunol. 2022;238:108996.

15. Więsik-Szewczyk E, Zegadło A, Sobczyńska-Tomaszewska A, Korzeniowska M, Jahnz-Rózyk K. Case report: VEXAS as an example of autoinflammatory syndrome in pulmonology clinical practice. Front Med (Lausanne). 2024;11:1340888.

16. Freitas CM, Silva ACP, Kloster NN, Speers AG, Neri ERM, Garcia MM, et al. Case report: Acute myeloid leukemia manifesting features of vexas syndrome in elderly woman. Hematol Transfus Cell Ther. 2023;45:S117–8.

17. Johnsson M. Rhombencephalitis in a patient with VEXAS syndrome. Neuroimmunology Reports. 2023;4:100176.

18. Raj S, Sadawarte S. MDS-432 challenges in diagnosis and treatment of VEXAS syndrome: Two case reports. Clin Lymphoma Myeloma Leuk. 2023;23:S366.

19. Zaimoku Y, Imi T, Hatada T, Mura H, Yoshino H, Tran DCC, et al. Prevalence and outcome of vexas syndrome in unrelated hematopoietic stem cell transplantation. Blood. 2023;142:1354–1354.

20. Al-Hakim A, Poulter JA, Mahmoud D, Rose AMS, Elcombe S, Lachmann H, et al. Allogeneic haematopoietic stem cell transplantation for VEXAS syndrome: UK experience. Br J Haematol. 2022;199:777–81.

21. 10 VEXAS, a vexing clinical case with renal involvement. Am J Kidney Dis. 2024;83:S3–4.

22. van Leeuwen-Kerkhoff N, de Witte MA, Heijstek MW, Leavis HL. Case report: Up-front allogeneic stem cell transplantation in a patient with the VEXAS syndrome. Br J Haematol. 2022;199:e12–5.

23. Ronsin C, Benard L, Mourtada A, Perrin F, Boukerroucha Z. Acute tubulointerstitial nephritis revealing VEXAS syndrome. Kidney Int. 2022;101:1295–7.

24. Nguyen JK, Routledge D, van Der Weyden C, Blombery P, Angel CM, Johnson D, et al. VEXAS syndrome: A dermatological perspective. Australas J Dermatol. 2022;63:488–92.

25. Tosato F, Pelloso M, Zuin J, Basso D. Peripheral blood cells vacuoles in VEXAS syndrome. Am J Hematol. 2023;98:1663–4.

26. Austestad J, Madland TM, Sandnes M, Haslerud TM, Benneche A, Reikvam H. VEXAS syndrome in a patient with myeloproliferative neoplasia. Case Rep Hematol. 2023;2023:6551544.

27. Abumanhal M, Leibovitch I, Zisapel M, Eviatar T, Edel Y, Ben Cnaan R. Ocular and orbital manifestations in VEXAS syndrome. EYE. 2024;38:1748–54.

28. Riescher S, Lecomte R, Danic G, Graveleau J, Le Bris Y, Hello M, et al. Susceptibility to mycobacterial infection in VEXAS syndrome. Rheumatology (Oxford) [Internet]. 2024; Available from: http://dx.doi.org/10.1093/rheumatology/keae087

29. Shimizu T, Ide H, Tsuji Y, Koga T, Kawakami A. VEXAS syndrome complicated with severe infection. Rheumatology (Oxford). 2022;61:e374–6.

30. Mayo-Juanatey A, Fernández-Llavador MJ, Valera-Ribera C, Valls-Pascual E, Alegre-Sancho JJ. Use of sarilumab in VEXAS syndrome. Rheumatology (Oxford) [Internet]. 2024; Available from: http://dx.doi.org/10.1093/rheumatology/keae068

31. Islam S, Cullen T, Sumpton D, Damodaran A, Heath D, Bosco A, et al. VEXAS syndrome: lessons learnt from an early Australian case series. Intern Med J. 2022;52:658–62.

32. Kao RL, Jacobsen AA, Billington CJ Jr, Yohe SL, Beckman AK, Vercellotti GM, et al. A case of VEXAS syndrome associated with EBV-associated hemophagocytic lymphohistiocytosis. Blood Cells Mol Dis. 2022;93:102636.

33. Miyoshi Y, Kise T, Morita K, Okada H, Imadome K-I, Tsuchida N, et al. Long-term remission of VEXAS syndrome achieved by a single course of CHOP therapy: A case report. Mod Rheumatol Case Rep. 2023;8:199–204.

34. R Pinto F, Lamas A, G Oliveira D, E Oliveira M, Faria R. VEXAS syndrome: A call for diagnostic awareness based on a case series of seven patients. Acta Med Port. 2023;36:379–80.

35. Wilson NR, Jain P, Gomez JA, Lu H, Pemmaraju N. Concurrent myelodysplasia and monoclonal B lymphocytosis in VEXAS syndrome. Leuk Res. 2022;120:106909.

36. Sofi FA, Naqati SM, Ahmad M, Bindroo M. VEXAS syndrome presenting as diffuse alveolar haemorrhage. BMJ Case Rep. 2024;17:e259474.

37. Amakusa Y, Suzuki T, Takemura M, Oguri T. Steroid reduction-resistant pulmonary involvement with Sweet’s syndrome suspected of being vacuoles, E1 enzyme, X-linked, autoinflammatory, somatic syndrome: A case report. Respirol Case Rep. 2024;12:e01288.

38. Delplanque M, Aouba A, Hirsch P, Fenaux P, Graveleau J, Malard F, et al. USAID associated with myeloid neoplasm and VEXAS syndrome: Two differential diagnoses of suspected adult onset Still’s disease in elderly patients. J Clin Med. 2021;10:5586.

39. Ciprian G. Adverse reaction to COVID-19 mRNA vaccination in a patient with VEXAS syndrome. Cureus. 2022;14:e23456.

40. Grey A, Cheong PL, Lee FJ, Abadir E, Favaloro J, Yang S, et al. A case of VEXAS syndrome complicated by hemophagocytic lymphohistiocytosis. J Clin Immunol. 2021;41:1648–51.

41. Suárez-Díaz S, Yllera-Gutiérrez C, Morán-Castaño C, Caminal-Montero L. Entities inside one another: VEXAS, a matryoshka-type disease. Reumatol Clín (Engl Ed). 2024;20:57–8.

42. Sharma A, Naidu G, Deo P, Beck DB. VEXAS syndrome with systemic lupus erythematosus: expanding the spectrum of associated conditions. Arthritis Rheumatol. 2022;74:369–71.

43. Salehi T, Callisto A, Beecher MB, Hissaria P. Tofacitinib as a biologic response modifier in VEXAS syndrome: A case series. Int J Rheum Dis. 2023;26:2340–3.

44. Kusne Y, Lasho T, Finke C, Patnaik MM, Badar T. VEXAS syndrome in a patient with DDX41 germline predisposition syndrome. Leuk Res. 2024;136:107432.
